# Paraneoplastic ocular syndromes: a systematic review of epidemiology, diagnosis and outcomes (2010–2023)

**DOI:** 10.1186/s12348-025-00534-1

**Published:** 2025-09-26

**Authors:** Solweig Beuzit, Aude Méal, Mathieu Delplanque, Jean-Christophe Ianotto, Béatrice Cochener-Lamard, Claire de Moreuil, Bénédicte Rouvière

**Affiliations:** 1 Department of Internal Medicine, University Hospital of Brest, Brest, France; 2 Department of Ophthalmology, University Hospital of Brest, Brest, France; 3 Department of Hematology, University Hospital of Brest, Brest, France; 4 University of Brest, INSERM, University Hospital of Brest, UMR 1304, GETBO, Brest, France; 5 University of Brest, INSERM, University Hospital of Brest, UMR 1227, B Lymphocytes and Autoimmunity, Brest, France

**Keywords:** Paraneoplastic ocular syndromes, Cancer-associated retinopathy (CAR), Melanoma-associated retinopathy (MAR), Bilateral diffuse uveal melanocytic proliferation (BDUMP), Paraneoplastic uveitis (PU), Paraneoplastic optic neuropathy (PON), Acute exudative polymorphous vitelliform maculopathy (AEPPVM)

## Abstract

**Background:**

Paraneoplastic ocular syndromes are rare, immune-mediated disorders triggered by malignancies. They may precede cancer diagnosis or signal its recurrence, highlighting their potential value as early warning signs. Their recognition is critical for timely diagnosis and appropriate management.

**Methods:**

We performed a systematic review of case reports and case series published between 2010 and 2023 in PubMed database, focusing on six major syndromes: cancer-associated retinopathy (CAR), melanoma-associated retinopathy (MAR), bilateral diffuse uveal melanocytic proliferation (BDUMP), acute exudative polymorphous paraneoplastic vitelliform maculopathy (AEPPVM), paraneoplastic uveitis (PU), and paraneoplastic optic neuropathy (PON). We extracted demographic, clinical, immunologic, oncologic, therapeutic, and outcome-related data.

**Results:**

A total of 132 articles comprising 147 patients were included: 53 with CAR, 22 with MAR, 26 with BDUMP, 16 with AEPPVM, 11 with PU, and 19 with PON. Visual impairment was bilateral in over 90% of cases. The most frequently associated malignancies were lung cancers (notably small-cell lung carcinoma), gynecological cancers, and melanoma. Onconeural autoantibodies were tested in serum—most commonly revealing anti-recoverin and anti-alpha-enolase in CAR, and anti-CRMP5 in PON—but were never assessed in cerebrospinal fluid (CSF), despite its potential diagnostic value. Therapeutic approach was highly heterogeneous and largely empirical, with systemic corticosteroids being the most commonly used treatment. Visual prognosis varied but was especially poor in CAR, for which 49.1% of patients experienced worsening vision. Notably, in CAR, an early oncologic diagnosis (within 6 months after symptom onset) was significantly associated with a favorable visual outcome (*p* = 0.03).

**Conclusion:**

We identified a clinical profile of patients in whom paraneoplastic ocular syndromes should be suspected. These rare inflammatory disorders may serve as early indicators of malignancy. Further studies are needed to improve diagnostic pathways, optimize immunologic workup (including CSF testing), and guide therapeutic strategies.

**Supplementary Information:**

The online version contains supplementary material available at 10.1186/s12348-025-00534-1.

## Introduction

Paraneoplastic syndromes are systemic manifestations associated with malignancies that are not directly attributable to local or metastatic tumor invasion, and that cannot be explained by nutritional deficiencies, metabolic disorders, infections or iatrogenic causes [1]. Paraneoplastic syndromes affect approximately 7 to 15% of cancer patients [2]. Bronchopulmonary cancers – especially small-cell lung carcinomas – are the most commonly associated malignancies. Although rare, these manifestations can be used as early markers of malignancy, and thus represent a major diagnostic challenge.

The term “masquerade syndrome” was first introduced in 1967 to describe a case of conjunctival carcinoma initially misdiagnosed as chronic and resistant conjunctivitis [3]. The concept was subsequently extended to a range of ocular pathologies which, by mimicking chronic idiopathic inflammation, can delay the identification of their underlying cause. In fact, these inflammatory manifestations correspond either to ocular neoplastic disorders (e.g. intraocular lymphoma or retinoblastoma), or to ocular paraneoplastic disorders [4]. This work focuses on the latter etiology.

Ocular paraneoplastic disorders were first described in 1976. They remain a rare entity, affecting only 0.01% of patients with malignancies [5]. Their rarity, associated with their polymorphic clinical presentation, makes their recognition and diagnosis particularly challenging.

The eye is theoretically protected from pathogenic inflammatory reactions to prevent significant loss of function due to tissue damage. In this respect, several mechanisms contribute to its immunological privilege, providing a state of immune tolerance. These include secretion of intraocular immunosuppressive factors, major histocompatibility complex class II low expression, and effector T cells conversion into regulatory T cells, Programmed Death-Ligand 1 and Fas-Ligand expression, which induce apoptosis of immune cells [6].

One explanation for the occurrence of ocular paraneoplastic disorders is based on the concept of “molecular mimicry”: a tumor may express some antigens similar to those naturally present in various organs, including the eye [7]. In this context, the host immune response, initially directed against the tumor, triggers the production of autoantibodies capable of recognizing these antigens in remote tissues, leading to potentially severe ophthalmic damage. One example is recoverin, a photoreceptor-specific protein involved in cancer-associated retinopathy, which may also be expressed in lung carcinoma cells [8].

Ocular paraneoplastic disorders can take different clinical forms. They represent an important warning signal and can sometimes precede the discovery of cancer, occur in parallel with its progression, or herald a recurrence.

This study aimed to provide a comprehensive description of these ophthalmic inflammatory phenomena associated with cancer through a systematic review of the literature between 2010 and 2023.

## Methods

The PubMed database was used to conduct a systematic literature review, using the keywords: “cancer-associated retinopathy”, “melanoma- associated retinopathy”, “paraneoplastic retinopathy” “bilateral diffuse uveal melanocytic proliferation”, “paraneoplastic vitelliform retinopathy”, “paraneoplastic acute exudative polymorphous vitelliform maculopathy”, “paraneoplastic uveitis”, “paraneoplastic optic neuropathy” and “paraneoplastic optic neuritis”. A single reviewer screened all abstracts retrieved to finalize article selection. The set of identified articles was cross-referenced with those retrieved from a search using the MESH term “paraneoplastic ocular syndromes “.

- Inclusion criteria were: clinical case descriptions and clinical case series, published between January 2010 and December 2023, with an available abstract, in French and English.
- Exclusion criteria were: general reviews, pathophysiology articles, exclusive therapeutic reviews, and pediatric cases.

Collected information included age, sex, age at diagnosis of the ophthalmic condition, age at oncological diagnosis, type of ophthalmic condition and associatedclinical and paraclinical data at diagnosis (visual acuity at diagnosis, slit-lamp examination, fundus examination, angiography, optical coherence tomography (OCT), and electroretinogram (ERG)), results of cerebrospinal fluid analysis, anterior chamber or vitreous biopsy, presence of circulating autoantibodies, brain magnetic resonance imaging (MRI), data on local or systemic ophthalmic treatments (corticosteroids, immunosuppressants, and biologic therapies), oncological and hematological diagnoses, their management (surgery, radiotherapy, chemotherapy, immunotherapy, targeted therapy), and outcomes after treatment regarding ophthalmic and oncological conditions.

The primary aim of this study was to describe the epidemiological characteristics of the population identified through the systematic literature review.

The secondary aims were to describe clinical data related to ophthalmic conditions, biological data focusing on autoantibodies, investigate the timeline between ophthalmic conditions and cancer diagnosis, describe immunosuppressive treatments used for ophthalmic conditions, and assess the evolution of ophthalmic conditions in relation to cancer progression.

Statistical analyses were performed using GraphPad Prism 5^®^ software. Percentages and frequencies of demographic, clinical, and biological variables were calculated for the entire study population as well as for the subgroup of patients with available clinical outcome data. When visual progression data were available, qualitative data comparisons between subgroups of patients with favorable and unfavorable progression were performed using the Chi-square test or Fisher’s exact test when the sample size was below five per group. Quantitative data comparisons between the two groups were conducted using the Student’s t-test or the Wilcoxon-Mann-Whitney exact test when the sample size was below five per group.

## Results

### Enrolled population

A literature search of the PubMed database identified 481 articles related to ocular paraneoplastic syndromes published between 2010 and 2023. Among these 481 articles, 155 focused on cancer associated retinopathy (CAR, 32.2%), 83 on melanoma associated retinopathy (MAR, 17.3%), 48 on acute exudative polymorphous paraneoplastic vitelliform maculopathy (AEPPVM, 10%), 94 on bilateral diffuse uveal melanocytic proliferation (BDUMP, 19.5%), 64 on paraneoplastic uveitis (PU, 13.3%), and 37 on paraneoplastic optic neuropathies (PON, 7.7%). Of the 481 articles initially identified, 287 were excluded (59.7%) according to predefined eligibility criteria: 58 were general reviews (20.2%), 20 were focused exclusively on therapeutics (7%), 36 were dedicated to pathophysiological mechanisms (12.5%), 155 described non-paraneoplastic inflammatory ophthalmic conditions (54%), 4 involved pediatric patients (1.4%), and 2 were duplicate reports (0.7%).

**Fig. 1:**
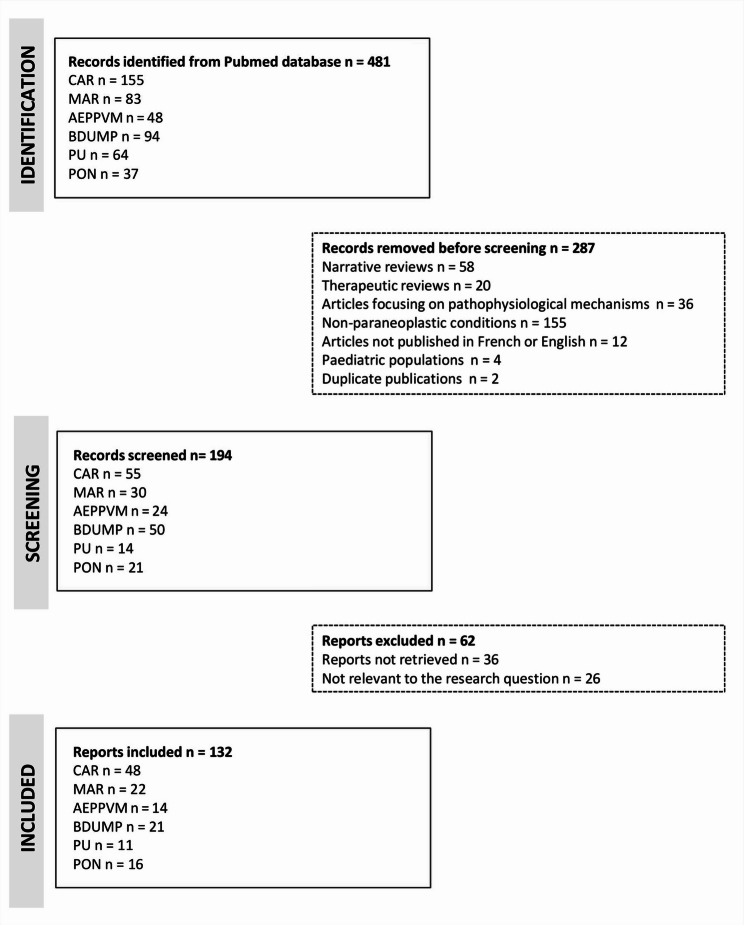
Flow chart of reports selected according to PRISMA guidelines. CAR: Carcinoma associated retinopathy, MAR: Melanoma associated retinopathy, AEPPVM: Acute exudative paraneoplastic polymorphous vitelliform maculopathy, BDUMP: Bilateral diffuse uveal melanocytic proliferation, PU: paraneoplastic uveitis, PON: paraneoplastic optic neuropathy.

Ultimately, from January 1, 2010, to December 31, 2023, 132 case reports or case series were included in this systematic literature review, comprising: 48 articles on CAR, 22 on MAR, 14 on AEPPVM, 21 on BDUMP, 11 on paraneoplastic uveitis, and 16 on paraneoplastic optic neuropathies (Fig. 1).

Details of the studies included in this review are provided in the appendix (Tables 5, 6, 7, 8, 9 and 10).

### Demographic data

There was no significant difference in mean age between the groups of patients according to each ophthalmic condition (*p* = 0.2) (Table 1). Mean ages were 61.9 years for CAR, 67.1 years for MAR, 66.5 years for BDUMP, 58.3 years for AEPPVM, 54.8 years for paraneoplastic uveitis, and 63.6 years for paraneoplastic optic neuropathies. Although no significant difference in sex was observed between the groups (*p* = 0.2), a trend was noted in some subgroups. In the MAR, BDUMP, and AEPPVM groups, males predominated, with male-to-female ratios of 2.1 (68.2% vs. 31.8%), 1.8 (65.4% vs. 34.6%), and 2.2 (68.7% vs. 31.3%), respectively.

**Table 1:** General and ophthalmic characteristics of the study population

	CAR *n* = 53	MAR *n* = 22	BDUMP *n* = 26	AEPPVM *n* = 16	PU *n* = 11	PON *n* = 19
Demographic characteristics						
Age (years)						
- Median (IQR)	65 (14)	68 (14.5)	64 (11.8)	58 (16.8)	59 (21)	67 (12)
- Mean (SD)	61.9 (12)	67.1 (9.2)	66.5 (8)	58.3 (12.6)	54.8 (19.2)	63.6 (11.4)
Sex						
-Female, *n* (%)	31 (58.5)	7 (31.8)	9 (34.6)	5 (31.3)	4 (36.4)	11 (52.6)
Country						
- Europe, *n* (%)	19 (35.8)	7 (31.8)	11 (42.3)	4 (25)	6 (54.5)	3 (15.8)
- North America, *n* (%)	16 (30.2)	9 (40.9)	2 (7.7)	11 (68.8)	1 (9.1)	5 (26.3)
- Asia, *n* (%)	15 (28.3)	5 (22.7)	11 (42.3)	-	1 (9.1)	10 (52.6)
- Oceania, *n* (%)	3 (5.7)	-	-	-	-	0
- Middle East, *n* (%)	-	1 (4.5)	1 (3.8)	-	3 (27.3)	1 (5.3)
- North Africa, *n* (%)	-	-	1 (3.8)	1 (6.3)	-	0
Ophthalmic characteristics						
Laterality						
- Bilateral, *n* (%)	49 (92.5)	21 (95.5)	24 (92.3)	15 (93.8)	8 (72.7)	15 (78.9)
- Unilateral, *n* (%)	4 (7.4)	1 (4.5)	2 (7.7)	1 (6.3)	3 (27.3)	4 (21.1)
Symptoms						
- Decreased visual acuity, *n* (%)	44 (83)	17 (77.3)	26 (100)	16 (100)	11 (100)	19 (100)
- Hemeralopia, *n* (%)	14 (26.4)	12 (54.5)	-	1 (6.3)	-	-
- Photopsia, *n* (%)	11 (20.8)	11 (50.0)	-	1 (6.3)	-	3 (15.8)
- Dyschromatopsia, *n* (%)	5 (9.4)	4 (18.2)	-	-	-	-
- Photophobia, *n* (%)	6 (11.3)	2 (9.1)	-	1 (6.3)	1 (9.1)	1 (5.3)
- Pain, *n* (%)	-	-	-	-	2 (18.2)	-
- Ocular redness, *n* (%)	-	-	-	-	3 (27.3)	-
- Visual field defect, *n* (%)	40 (75.5)	14 (63.6)	-	-	-	1 (5.3)
Uveitis, *n* (%)	7 (13)	5 (22.7)	2 (7.7)	1 (6.3)	11 (100)	7 (36.8)
- Anterior, *n* (%)	1 (14.3)	-	2 (7.7)	1 (6.3)	3 (27.3)	-
- Intermediate, *n* (%)	1 (14.3)	5 (22.7)	-	-	3 (27.3)	7 (100)
- Posterior, *n* (%)	-	-	-	-	3 (27.3)	-
- Panuveitis, *n* (%)	5 (71.4)	-	-	-	2 (18.2)	-
Retinal vasculitis, *n* (%)	13 (24.5)	-	-	-	1 (9.1)	-
OCT Findings, *n* (%)	29 (54.7)	6 (27.3)	25 (96.2)	16 (100)	5 (45.5)	6 (31.6)
- Serous retinal detachment, *n* (%)	1 (3.4)	-	17 (68)	11 (68.8)	1 (9.1)	3 (50)
- Ellipsoid zone disruption, *n* (%)	11 (37.9)	1 (16.7)	1 (4)	1(6.3)	1 (9.1)	1 (16.7)
- Outer photoreceptor segment involvement, *n* (%)	11 (37.9)	-	-	1(6.3)	-	-
- Macular oedema, *n* (%)	3 (10.3)	-	-	-	-	-
- Retinal atrophy, *n* (%)	2 (6.9)	3 (50)	-	-	-	3 (33.3)
- Alternating RPE thickening/atrophy, *n* (%)	-	-	9 (36)	-	-	-
- Subretinal deposits, *n* (%)	-	-	-	9 (56.3)	-	-
- Others, *n* (%)	2 (6.9)	2 (33.3)	4 (16)	1 (6.3)	3 (27.3)	-
Additional investigations						
ERG, *n* (%)	42 (79.2)	22 (100)	1 (3.8)	8 (50)	-	-
- Combined cone and rod dysfunction, *n* (%)	35 (83.3)	13 (59.1)	1 (3.8)	4 (50)	-	-
- Isolated cone dysfunction, *n* (%)	6 (14.3)	-	-	-	-	-
- Isolated rod dysfunction, *n* (%)	1 (2.4)	9 (40.9)	-	-	-	-
Motor Evoked Potentials, *n* (%)	-	-	-	-	-	8 (42.1)
- Prolonged latency, *n* (%)	-	-	-	-	-	5 (62.5)
- Reduced amplitude, *n* (%)	-	-	-	-	-	3 (37.5)
Brain MRI						
- Not performed, *n* (%)	34 (64.2)	10 (45.5)	24 (92.3)	16 (100)	2 (18.2)	11 (57.9)
- Normal, *n* (%)	17 (32.1)	12 (54.5)	1 (3.8)	-	8 (72.7)	2 (10.5)
- Abnormal, *n* (%)	2 (3.8)	-	(3.8)	-	1 (9.1)	6 (31.6)

*AEPPVM *Acute exudative polymorphous paraneoplastic vitelliform maculopathy, *BDUMP *Bilateral diffuse uveal melanocytic proliferation, *CAR *Cancer-associated retinopathy, *ERG *Electroretinogram, *MAR * Melanoma-associated retinopathy, *MRI * Magnetic resonance imaging, *PON *Paraneoplastic optic neuropathy, *OCT *Optical coherence tomography, *PU* Paraneoplastic uveitis, *RPE* Retinal pigment epithelium

### Ophthalmic clinical data

Across all pathologies, bilateral ophthalmic involvement was present in the vast majority of patients (> 90%). Among patients with paraneoplastic uveitis and paraneoplastic optic neuropathy, involvement was also predominantly bilateral, but to a lesser extent, at 72.7% and 78.9%, respectively.

Decreased visual acuity was the most common symptom, observed in 100% of patients with BDUMP, AEPPVM, paraneoplastic uveitis and paraneoplastic optic neuropathy, in 83% of patients with CAR, and to a lesser extent in patients with MAR (77.3%). Half of the patients with MAR also reported hemeralopia and photopsia. Visual field defects were present in 75.5% of patients with CAR and 63.6% of those with MAR.

Among patients with CAR, 13% had associated uveitis, which was panuveitis in over 70% of these cases. In patients with paraneoplastic optic neuropathy, 36.8% also presented with uveitis, all of which were isolated intermediate uveitis. Retinal vessel vasculitis, predominantly venous vasculitis, was observed in 24.5% of patients with CAR and in 9.1% of those with paraneoplastic uveitis. In addition, involvement of the outer segment of the photoreceptors and of the ellipsoid zone on OCT was noted in 37.9% of patients with CAR.

A serous retinal detachment, consistent with the relevant pathologies, was observed in 68% of patients with BDUMP and in 68.8% of those with AEPPVM. Mixed cone and rod involvement on ERG was more frequently found in patients with CAR than in those with MAR (83.3% vs. 59.1%), whereas isolated rod involvement on ERG was more commonly observed in patients with MAR than in those with CAR (40.9% vs. 2.4%).

Brain MRI was rarely performed across all paraneoplastic conditions and was mostly normal when conducted, except in cases of paraneoplastic optic neuropathy, where 31.6% of patients showed inflammatory signals involving the optic nerve (Table 1).

### Biological data

The search for serum onconeural autoantibodies was mainly conducted in patients with CAR, MAR, and paraneoplastic optic neuropathy, with testing rates of 71.7%, 72.7%, and 94.7%, respectively. On the other hand, it was less frequently performed in patients with paraneoplastic uveitis, with a testing rate of 27.3%. Antibody testing in the cerebrospinal fluid has never been performed. In patients with CAR, anti-alpha-enolase antibodies (42.9%) and anti-recoverin antibodies (32.1%) were the most commonly detected. In patients with MAR, anti-TRPM-1 antibodies were the most frequently identified (35.7%). Among patients with paraneoplastic optic neuropathy, anti-CRMP5 antibodies were found in 92.3% of cases. Lumbar puncture usage varied according to the underlying pathology. It was performed in only 13% of patients with CAR and not at all in those with MAR, BDUMP, or AEPPVM. In contrast, lumbar puncture was more frequently performed in patients with paraneoplastic uveitis and paraneoplastic optic neuropathy with respective rates of 36.4% and 57.9%. In cases of paraneoplastic uveitis, cerebrospinal fluid (CSF) analysis was always normal when it was performed. In patients with paraneoplastic optic neuropathy, around one-third of the patients presented increased CSF protein. (Table 2)

**Table 2:** Biological characteristics of the study population

	CAR *n* = 53	MAR *n* = 22	BDUMP *n* = 26	AEPPVM *n* = 16	PU *n* = 11	PON *n* = 19
Serum antibodies						
Not performed, *n* (%)	15 (28.3)	6 (27.3)	26 (100)	11 (68.8)	7 (63.6)	1 (5.3)
Positive, *n* (%)	28 (52.8)	14 (63.6)	-	4 (25)	3 (27.3)	13 (68.4)
- Recoverin, *n* (%)	9 (32.1)	-	-	-	-	-
- Alpha-enolase, *n* (%)	12 (42.9)	2 (14.3)	-	-	-	-
- Alpha-aldolase, *n* (%)	2 (7.1)	2 (14.3)	-	-	-	-
- TRPM1, *n* (%)	2 (7.1)	5 (35.7)	-	-	-	-
- GAPDH, *n* (%)	6 (21.4)	-	-	-	-	-
- CRMP5, *n* (%)	3 (10.7)	-	-	-	3 (100)	12 (92.3)
- IRBP, *n* (%)	-	-	-	1 (6.3)	-	-
- PRDX3, *n* (%)	-	-	-	1 (6.3)	-	-
- Amphiphysin, *n* (%)	-	-	-	-	-	-
- Others, *n* (%)	7 (25)	11 (78.6)	-	2 (12.5)	-	5 (26.3)
Negative, *n* (%)	10 (18.9)	2 (9.1)	-	1 (6.2)	1 (9.1)	5 (26.3)
Lumbar puncture						
Not performed, *n* (%) Normal, *n* (%) Increased CSF protein, *n* (%) CSF lymphocytosis, *n* (%)	46 (86.8) 3 (5.7) 3 (5.7) 1 (1.8)	22 (100)	26 (100)	16 (100)	7 (63.6) 4 (36.4)	8 (42.1) 5 (26.3) 6 (31.6)

*AEPPVM* Acute exudative polymorphous paraneoplastic vitelliform maculopathy, *BDUMP* Bilateral diffuse uveal melanocytic proliferation, *CAR* Cancer-associated retinopathy, *CRMP5* Collapsin response mediator protein 5,* CSF *Cerebrospinal fluid, *GAPDH *Glyceraldehyde-3-phosphate dehydrogenase, IRBP: Interphotoreceptor retinoid-binding protein, *MAR* Melanoma-associated retinopathy, *PON* Paraneoplastic optic neuropathy, *PRDX3* Peroxiredoxin 3, *PU *Paraneoplastic uveitis, *TRPM1 *Transient receptor potential cation channel subfamily M member 1

### Oncological data

CAR was predominantly associated with pulmonary cancers (32.1% of cases, 82.4% of which were small cell lung carcinomas) and gynecological cancers (30.2%). At the time of ophthalmic diagnosis, 15% of patients with CAR had evidence of metastatic disease. By definition, 100% of cancers associated with MAR are melanomas. At the time of the ophthalmic diagnosis, 68.2% of patients had metastatic melanoma, and 31.8% had a recurrence which was metastatic in over 90% of cases.

In patients with BDUMP, the most commonly associated cancer type was also lung cancer (42.3%), followed by gynecological cancers (19.2%) and urological cancers (19.2%). At the time of the ophthalmic diagnosis, metastatic involvement was present in 38.5% of cases.

In patients with AEPPVM, melanomas were the most frequently associated cancers (50%), followed by multiple myelomas (31.3%). At the time of the ophthalmic diagnosis, 62.5% of patients (with non-hematologic cancers) had metastatic involvement.

Paraneoplastic uveitis was predominantly associated with pulmonary cancers (36.4%) and lymphomas (27.3%). At the time of the ophthalmic diagnosis, the malignancy was metastatic in 27.3% of cases.

In patients with paraneoplastic optic neuropathy, lung cancer was also the most frequently diagnosed cancer, affecting 63.9% of patients. At the time of the ophthalmic diagnosis, the oncological involvement was metastatic in 31.6% of cases.

In most cases, the oncological diagnosis was made after the onset of visual defect, except in patients with BDUMP and AEPPVM, where the underlying malignancy was already known in respectively 42.3% and 43.8% of cases at the time of ophthalmic involvement. The neoplasm was identified following the ophthalmic diagnosis in 81.1% of patients with CAR, 59.1% with MAR, 57.7% with BDUMP, 100% with paraneoplastic uveitis, and 78.9% with paraneoplastic optic neuropathy. (Table 3)

**Table 3:** Descriptions of neoplasms, treatments, visual and oncological outcomes

	CAR *n* = 53	MAR *n* = 22	BDUMP *n* = 26	AEPPVM *n* = 16	PU *n* = 11	PON *n* = 19
Neoplasms						
Lung, *n* (%)	17 (32.1)	-	11 (42.3)	1 (6.3)	4 (36.4)	12 (63.9)
- NSCLC, *n* (%)	14 (82.4)	-	6 (54.5)	-	4 (100)	8 (66.7)
- Other, *n* (%)	3 (17.6)	-	5 (45.5)	1 (6.3)	-	4 (33.3)
Gynecological, *n* (%)	17 (30.2)	-	5 (19.2)	-	1 (9.1)	1 (5.3)
Digestive, *n* (%)	6 (11.3)	-	2 (7.7)	-	-	2 (10.5)
Urological, *n* (%)	7 (13.2)	-	5 (19.2)	-	1 (9.1)	1 (5.3)
Other solid neoplasms, *n* (%)	5 (9.4)	-	2 (7.7)	1 (6.3)	2 (18.2)	2 (10.5)
Lymphoma, *n* (%)	2 (3.8)	-	1 (3.8)	1 (6.3)	3 (27.3)	-
Myeloma, *n* (%)	-	-	-	5 (31.3)	-	-
Other hematologic diseases, *n* (%)	-	-	-	-	-	-
Melanoma, *n* (%)	-	22 (100)	-	8 (50)	-	2 (10.5)
Metastatic, *n* (%)	8 (15.1)	15 (68.2)	10 (38.5)	10 (62.5)	3 (27.3)	6 (31.6)
Recurrence, *n* (%)	-	7 (31.8)	-	-	-	-
Diagnostic delay						
Oncologic diagnosis prior to visual involvement, *n* (%)	10 (18.9)	9 (40.9)	11 (42.3)	7 (43.8)	-	4 (21.1)
- Recurrence/Progression, *n* (%)	2 (20)	7 (31.8)	2 (18.2)	-	-	-
Oncologic diagnosis after visual involvement, *n* (%)	43 (81.1)	13 (59.1)	15 (57.7)	8 (50)	11 (100)	15 (78.9)
- < 6 months, *n* (%)	39 (90.7)	11 (84.6)	14 (93.3)	5 (62.5)	9 (81.8)	12 (80)
− 6 to 12 months, *n* (%)	2 (4.7)	-	-	1 (12.5)	2 (18.2)	3 (20)
- >12 months, *n* (%)	2 (4.7)	2 (4.7)	1 (6.7)	2 (25)	-	-
Unknown, *n* (%)	-	-	-	1 (6.3)	-	-
Immunosuppressive treatments						
No, *n* (%)	16 (30.2)	13 (59.1)	15 (57.7)	3 (18.8)	1 (9.1)	2 (10.5)
Yes, *n* (%)	37 (69.8)	7 (31.8)	9 (34.6)	7 (43.8)	10 (90.9)	12 (63.2)
- Systemic corticosteroids, *n* (%)	11 (29.7)	6 (85.7)	3 (33.3)	6 (85.7)	10 (100)	12 (100)
- Intravenous immunoglobulins, *n* (%)	9 (24.3)	1 (14.3)	-	-	-	1 (8.3)
- Plasmapheresis, *n* (%)	5 (13.5)	-	7 (77.8)	1 (14.3)	-	3 (25)
- Mycophenolate mofetil, *n* (%)	3 (8.1)	-	-	-	-	2 (16.7)
- Azathioprine, *n* (%)	3 (8.1)	-	-	-	-	-
- Ciclosporin, *n* (%)	2 (5.4)	-	-	-	-	-
- Rituximab, *n* (%)	3 (8.1)	-	-	-	-	-
- Anti TNF-alpha, *n* (%)	-	-	-	-	-	-
- Cyclophosphamide, *n* (%)	1 (2.7)	-	-	1 (4.3)	-	-
- Lenalidomide/Thalidomide, *n* (%)	-	-		4 (57.1)	-	-
Unknown, *n* (%)	-	2 (9.1)	2 (7.7)	6 (37.5)	-	5 (26.3)
Visual outcome						
Recovery, *n* (%)	5 (9.4)	4 (18.2)	3 (11.5)	6 (37.5)	7 (63.6)	1 (5.3)
Improvement, *n* (%)	14 (26.4)	10 (45.5)	7 (26.9)	2 (12.5)	1 (9.1)	7 (36.8)
Stability, *n* (%)	10 (18.9)	2 (9.1)	4 (15.4)	2 (12.5)	-	2 (10.5)
Worsening, *n* (%)	16 (30.2)	4 (18.2)	6 (23.1)	1 (6.3)	1 (9.1)	3 (15.8)
Unknown, *n* (%)	8 (15.1)	2 (9.1)	6 (23.1)	5 (31.25)	2 (18.2)	6 (31.6)
Oncological outcome						
Recovery, *n* (%)	14 (26.4)	6 (27.3)	2 (7.7)	3 (18.8)	5 (45.5)	1 (5.3)
Improvement, *n* (%)	4 (7.5)	1 (4.5)	2 (7.7)	4 (25)	2 (18.2)	-
Stability, *n* (%)	2 (3.8)	1 (4.5)	1 (3.8)	-	-	-
Worsening, *n* (%)	2 (3.8)	-	5 (19.2)	-	1 (9.1)	1 (5.3)
Death, *n* (%)	7 (13.2)	3 (13.6)	4 (15.4)	2 (12.5)	-	5 (26.3)
Unknown, *n* (%)	24 (45.3)	11 (50)	12 (75)	7 (43.8)	3 (27.3)	12 (63.2)

* AEPPVM* Acute exudative polymorphous paraneoplastic vitelliform maculopathy, *BDUMP *Bilateral diffuse uveal melanocytic proliferation, *CAR* Cancer-associated retinopathy, *MAR *Melanoma-associated retinopathy, *NSCLC *Non-small cell lung cancer, *PON* Paraneoplastic optic neuropathy, *PU* Paraneoplastic uveitis, *TNF* Tumor necrosis factor

### Therapeutic data

Immunosuppressive treatments were initiated to manage ophthalmic involvement in 69.8% of patients with CAR, 31.8% with MAR, 34.6% with BDUMP, 43.8% with AEPPVM, 90.9% with paraneoplastic uveitis, and 63.2% with paraneoplastic optic neuropathy. Systemic corticosteroids were the most commonly administered treatment.

All patients with paraneoplastic uveitis and all those with paraneoplastic optic neuropathy, when they were treated, received systemic corticosteroids. Among treated patients with MAR, 85.7% received either oral or intravenous corticosteroids. In patients with CAR, 29.7% of treated patients received systemic corticosteroids, and 24.3% received intravenous immunoglobulin (IVIg) at immunomodulatory doses.

Among those treated for BDUMP, 77.8% underwent plasmapheresis and 33.3% received systemic corticosteroids.

In the AEPPVM group, 85% of treated patients received systemic corticosteroids, and 57.1% received immunosuppressive treatment related to their underlying myeloma. (Table 3)

### Visual prognosis

Visual prognosis was assessed based on the evolution of visual acuity, with a favorable outcome defined as either complete recovery or improvement, and an unfavorable outcome defined as stable or worsening visual impairment.

In the group of patients with CAR, 49.1% had an unfavorable visual outcome, while 35.8% had a favorable one. Visual outcome was unavailable in 15.1% of cases. No correlation was observed between favorable visual prognosis and age, sex, type of visual symptoms, or the presence of retinal vasculitis. Immunosuppressive treatment, autoantibody positivity, presence of metastases at diagnosis, and the type of associated cancer were not predictive either.

However, an early oncological diagnosis—within six months of ophthalmic symptoms onset—was significantly associated with a favorable visual outcome (*p* = 0.03). (Table 4)

**Table 4:** Visual prognosis in subgroups patients with CAR

	Favorable visual outcome *n* = 19	Unfavorable visual outcome *n* = 26	*p* =
Mean age, years (SD)	64 (8.3)	62 (13)	0.8
Median age, years (IQR)	65 (12)	65 (14)	0.8
Female, *n* (%)	11 (57.9)	16 (61.5)	0.8
Visual acuity loss, *n* (%)	16 (84.2)	23 (88.5)	0.7
Visual field defect, *n* (%)	14 (73.2)	19 (73.1)	0.9
Retinal vasculitis, *n* (%)	4 (21.1)	6 (23.1)	0.9
Immunosuppressive treatment, *n* (%)	14 (73.7)	16 (61.5)	0.5
Corticosteroids, *n* (%)	13 (68.4)	15 (57.7)	0.5
Oncologic diagnosis < 6 months after visual involvement, *n* (%)	17 (89.5)	16 (61.5)	0.03
Serum autoantibodies, *n* (%)	8 (42.1)	13 (50)	0.6
Presence of metastases, *n* (%)	4 (21.1)	4 (15.4)	0.7
Pulmonary neoplasm, *n* (%)	9 (47.4)	9 (34.6)	0.4
Gynecological neoplasm, *n* (%)	5 (26.3)	9 (34.6)	0.9

In the MAR group, 63.6% of patients experienced a favorable visual outcome, while 27.3% had an unfavorable outcome. No clinical or biological characteristic appeared to be associated with visual prognosis in patients with MAR. Among patients with BDUMP, half experienced a favorable visual outcome and the other half an unfavorable one. In the AEPPVM group, 50% of patients showed a favorable visual outcome, while 18.8% had an unfavorable outcome. For patients with paraneoplastic uveitis, 63.7% had a favorable outcome and 9.1% an unfavorable one. Finally, in patients with paraneoplastic optic neuropathy, 42.1% experienced a favorable visual outcome, while 26.3% had an unfavorable evolution. (Table 3)

## Discussion

In this systematic review of inflammatory ocular paraneoplastic syndromes, we identified and analyzed 147 patients. These included 53 patients with CAR (36.1%), 22 with MAR (15%), 26 with BDUMP (17.7%), 16 with AEPPVM (10.9%), 11 with paraneoplastic uveitis (7.5%) and 19 with paraneoplastic optic neuropathy (12.9%).

This review highlights the rarity of these conditions and raises the possibility of underdiagnosis. To date, no systematic review has covered the full spectrum of paraneoplastic ocular syndromes. However, a UK-based review focusing solely on CAR between 1984 and 2022 identified only 28 patients [9].

The median age across these syndromes ranged from 58 to 68 years, with an overall median age of 64.5 years. These figures closely align with the median age at cancer diagnosis in France in 2023—70 years for men and 68 years for women [10]. Regarding sex distribution, the slight female predominance in CAR cases is consistent with existing literature [11]. In contrast, a higher proportion of males was observed among patients with MAR and AEPPVM, likely due to the high incidence of melanoma in these groups (100% in MAR, 50% in AEPPVM). This trend may also reflect the predominance of cases originating from North America, where melanoma’s incidence is higher in men [12]. A male predominance was also observed in BDUMP, consistent with literature reports, possibly related to the higher incidence of lung cancer among men [1].

Geographically, most patients originated from Europe, North America, or Asia (mainly Japan). There was a clear underrepresentation of cases from Africa and South America, likely due to limited healthcare access and diagnostic limitations in those regions.

In the majority of cases, paraneoplastic ocular manifestations were bilateral and resulted in significant visual acuity loss. CAR and MAR patients presented with more varied symptoms, including photopsia, photophobia, and dyschromatopsia. Night blindness was also more common in MAR, consistent with bipolar ON cell involvement, which is responsible for rod signal transmission. Visual impairment appeared to be less severe in MAR, in line with previously published data [1]. Apart from characteristic ophthalmic presentations such as BDUMP and AEPPVM, the clinical symptomatology is often nonspecific, making diagnosis based solely on ophthalmic findings particularly challenging.

Cerebrospinal fluid analysis was rarely performed, across all syndromes. Its diagnostic utility depends on the specific condition: in BDUMP and AEPPVM, where fundoscopic findings are characteristic, lumbar puncture offers limited additional information. In contrast, in cases like CAR, MAR, uveitis, and optic neuropathies, lumbar puncture may provide valuable diagnostic insight, although no formal guidelines currently recommend its routine use [13]. In uveitis workups, lumbar puncture is reserved for ruling out central nervous system inflammation, infectious etiologies, or ocular lymphoma [14]. In our clinical practice, it is a third-line investigation in idiopathic ocular inflammation. When performed, cerebrospinal fluid analysis revealed elevated protein levels in nearly half of cases, indicating central nervous system inflammation. However, autoantibody testing in CSF was never conducted. In other paraneoplastic conditions like encephalitis, onconeural antibody detection in cerebrospinal fluid has a positivity rate between 74% and 93%, making it diagnostically useful [15]. Moreover, antibody detection sensitivity varies between serum and CSF: onconeural antibodies are more reliably identified in serum, while CSF analysis is more informative for detecting surface antigen antibodies [16]. Serum testing for onconeural autoantibodies was more frequently performed in CAR, MAR, and paraneoplastic optic neuropathy. Among CAR patients, anti-alpha enolase antibodies were the most commonly detected (42.9%), consistent with literature reporting positivity in approximately one-third of cases. Anti-recoverin antibodies were found in 32.1% of patients, compared to 10% in the literature [2, 6]. In our study, anti-CRMP5 antibodies predominated among paraneoplastic optic neuropathies (92.3%). Several studies have linked anti-CRMP5 antibodies to paraneoplastic manifestations, mainly neurological syndromes [17]. Peripheral neuropathies are the most common presentation, but ocular involvement—most often paraneoplastic optic neuropathy, occasionally with retinitis or uveitis—has also been reported [18]. Importantly, only 20% of patients with anti-CRMP5 antibodies develop PON, highlighting the limited specificity of this biomarker for paraneoplastic ophthalmic syndromes [19]. More broadly, the clinical utility of autoantibody testing in suspected autoimmune retinopathies—whether paraneoplastic or not—is constrained by significant limitations. Antiretinal antibodies have been detected in non-autoimmune ocular diseases (e.g., anti-recoverin in cataract), in systemic disorders without ocular involvement, and even in healthy individuals [20, 21]. Furthermore, inter-laboratory variability is considerable. In one comparative study, overall concordance for antiretinal antibody detection was only 57%. Notably, 93% of patients without autoimmune retinopathy were tested positive by Western blot and 86% by immunohistochemistry. While none demonstrated anti-recoverin antibodies, 64% showed anti-α-enolase antibodies, yielding an estimated specificity of only 7% (95% CI: 0–34%) [22]. In addition, a negative serology does not exclude the diagnosis—approximately 20% of CAR and PON cases were seronegative in our review.

Taken together, these findings show that antibody testing should serve as an adjunctive tool and be interpreted only in the appropriate clinical context—such as rapidly progressive bilateral vision loss with characteristic OCT and ERG abnormalities—rather than as a confirmatory diagnostic criterion. Their main value lies in reinforcing clinical suspicion in a compatible setting, particularly in older patients (> 60 years) with suspected paraneoplastic ophthalmic syndromes, where both serum and CSF testing may be considered.

Most associated cancers were pulmonary, predominantly small-cell lung carcinomas. This finding is consistent with the literature, as lung cancer is the most common neoplasm in paraneoplastic syndromes [23], and lung tumors can express recoverin or α-enolase—also found in photoreceptors [8]. AEPPVM was an exception, with melanoma being the most frequently associated cancer, in line with previously reported data [24]. Interestingly, 31% of AEPPVM patients had multiple myeloma—a novel finding. This may reflect an immune response to monoclonal immunoglobulins from neoplastic plasma cells. In most cases, cancer was diagnosed simultaneously or within six months of the onset of ocular symptoms, consistent with prior reports [25]. However, while MAR is traditionally described in the context of known melanoma, only 41% of patients in our series had a prior melanoma diagnosis. Among them, 68.2% presented with metastatic recurrence. Apart from MAR, cancers were typically diagnosed at limited stages, with metastatic disease found in only 15.1–38.5% of cases.

These findings highlight the internist’s role in diagnosing these rare syndromes. A paraneoplastic hypothesis should be considered in patients over 50 presenting with bilateral idiopathic ocular inflammation and visual decline, especially with cancer risk factors (smoking, alcohol use, environmental exposures). Early diagnosis enables treatment of the neoplasm at a potentially curable stage. Chest computed tomography (CT) is already recommended in adults over 50 with chronic non-infectious uveitis to exclude sarcoidosis [26]. This exam could also help screen for lung cancer. If uninformative, positron emission tomography-CT may be warranted to rule out occult malignancy.

There is currently no consensus regarding treatment. Systemic corticosteroids were the most commonly used immunosuppressive treatment, despite limited high-quality evidence supporting their efficacy [1]. Notably, 25 patients (17%) achieved favorable visual outcomes without receiving any immunosuppressive treatment—5 with CAR, 10 with MAR, 6 with BDUMP, 2 with AEPPVM, 1 with paraneoplastic uveitis treated locally, and 1 with paraneoplastic optic neuropathy. Among patients with CAR, 69.8% underwent immunosuppressive treatment, yet only 29.7% received systemic corticosteroids—a lower proportion compared to other syndromes. Existing literature suggests that systemic steroids provide, at best, a modest visual benefit [27]. In MAR, the absence of immunosuppressive treatment in 60% of cases may be attributed to the relatively milder disease course and a favorable response to melanoma treatment. In BDUMP, more than half of the treated patients underwent plasmapheresis, likely reflecting the presumed role of circulating factors such as CMEP (cultured melanocyte elongation and proliferation factor) [28]. Beyond corticosteroids, immunosuppressive strategies varied widely. Combining immunosuppressive treatments with chemotherapy represents a major challenge due to the increased risk of treatment-related toxicity (neutropenia, anemia, and other side effects). Moreover, the contraindication of anti-TNF alpha agents—which remain a first-line treatment for chronic non-infectious uveitis—in cases of active cancer complicates therapeutic decision-making. The use of newer therapies for chronic non-infectious uveitis, such as tocilizumab, may offer a promising alternative in paraneoplastic uveitis and, by extension, in other paraneoplastic ocular inflammatory conditions [29, 30].

CAR was frequently associated with a poor visual prognosis: 50% of patients worsened versus 35.8% improved. The evolution of the disease appeared independent of cancer control, and immunosuppressive therapies often failed to improve vision. In our series, early cancer diagnosis and prompt oncologic treatment were the only factors associated with better visual outcomes. In BDUMP, visual outcomes were mixed. Plasmapheresis appeared correlated with improvement. Other paraneoplastic eye conditions generally had more favorable visual outcomes, often better than their oncologic prognosis. In MAR, favorable visual outcomes may reflect effective melanoma treatment with visual improvement closely linked to tumor burden reduction [11]. In our review, among patients with available oncologic status, outcomes were favorable in 31.8% (recovery and improvement). This association may extend to AEPPVM, where melanoma was also the predominant cancer. In paraneoplastic uveitis, the favorable visual outcome may also be explained by the favorable oncological evolution observed in more than 60% of the patients. In addition, the well-established efficacy of systemic corticosteroids in uveitis, which remain the first-line treatment [31]—used in 90% of our cases—likely contributed to these results. Similarly, patients with paraneoplastic optic neuropathy also showed favorable visual outcomes, likely due to corticosteroid response, as 63% received such treatment.

Our study has some limitations. The main limitation lies in the small sample sizes within each subgroup, most include fewer than 30 patients, with the exception of CAR (*n* = 53). This reduced the statistical power of our analysis. These small numbers stem from the rarity of these conditions (estimated at 0.01% of cancer patients) [5]. Secondly, there was substantial missing data. In literature-derived cases, oncologic outcome data were incomplete in 14–75% of cases; visual outcome data were missing in 14–30%. A multicenter prospective study could help to increase the sample size and reduce data gaps.

Furthermore, our study also presents several strengths. This is the first systematic review to comprehensively address all paraneoplastic ocular syndromes. The long inclusion period (2010–2023) allows for meaningful follow-up, enabling the assessment of both visual and oncological outcomes.

While not without limitations, this approach provides a broad and detailed understanding of the management of these rare conditions. This knowledge is crucial, as it may facilitate earlier oncological diagnosis and help prevent the inappropriate use of immunosuppressive therapies, which could potentially promote tumor progression.

## Conclusion

Paraneoplastic ocular syndromes, although rare and under-researched, represent a significant diagnostic challenge due to their potential role as early indicators of cancer or cancer recurrence. Early recognition may allow for faster cancer diagnosis and better visual prognosis. In this context, this study presents a systematic review of the scientific literature from 2010 to 2023.

These findings help define the clinical contexts in which paraneoplastic syndromes should be suspected, and highlight the diagnostic value of a multidisciplinary approach.

In conclusion, this work lays a foundation for improving the understanding and management of these syndromes. It emphasizes the importance of early diagnosis and multidisciplinary care. It also advocates for structured initiatives—such as national patient registries—to refine diagnostic and therapeutic strategies. Enhancing patient outcomes will require better care coordination and continued clinical research on these rare but revealing conditions.

## Supplementary Information

Supplementary Material 1.

Supplementary Material 2.

Supplementary Material 3.

Supplementary Material 4.

Supplementary Material 5.

Supplementary Material 6.

## Data Availability

All data generated or analyzed during this study are included in this published article and its supplementary information files.

## Abbreviations

AEPPVM: Acute exudative polymorphous paraneoplastic vitelliform maculopathy
BDUMP: Bilateral diffuse uveal melanocytic proliferation
CAR: Cancer-associated retinopathy
CMEP: cultured melanocyte elongation and proliferation factor
CRMP5: Collapsin response mediator protein 5
CSF: Cerebrospinal fluid
CT: Computed tomography
ERG: Electroretinogram
GAPDH: Glyceraldehyde-3-phosphate dehydrogenase
IVIg: Intravenous Immunoglobulin
IRBP: Interphotoreceptor retinoid-binding protein
MAR: Melanoma-associated retinopathy
MESH: Medical Subject Headings
MRI: Magnetic resonance imaging
NSCLC: Non-small cell lung cancer
OCT: Optical coherence tomography
PON: Paraneoplastic optic neuropathy
PRDX3: Peroxiredoxin 3
PU: Paraneoplastic uveitis
RPE: Retinal pigment epithelium
TNF alpha: Tumor necrosis factor alpha
TRPM1: Transient receptor potential cation channel subfamily M member 1
UK: United Kingdom
