# Supplementary material for: Paraneoplastic ocular syndromes: a systematic review of epidemiology, diagnosis and outcomes (2010–2023)
Source: J Ophthalmic Inflamm Infect. 2025 Sep 26;15:73. doi: 10.1186/s12348-025-00534-1 (PMC12474834; doi:10.1186/s12348-025-00534-1)
Supplement: Supplementary file 1 — Supplementary Material 1. [file 12348_2025_534_MOESM1_ESM.docx]

**Table 5: Characteristics of reported cases of cancer associated retinopathy.**

| **Author, year, country** | **Sex, age** | **Ophthalmologic data** | **Ophthalmologic exams** | **Systemic workup** | **Treatment** | **Cancer, diagnosis timing** | **Visual outcome** | **Cancer outcome** |
| --- | --- | --- | --- | --- | --- | --- | --- | --- |
| Z. Javaid, 2015, United Kingdom | F, 30 | **Lat:** Unilateral **Sx:** Decreased visual acuity, photopsia **VF:** Decreased **ACI:** No **Fundus exam:** Optic disc atrophy | **OCT:** N/A **FA:** Vascular hypofluorescence **ERG:** Pathologic (cones and rods) | **Brain MRI:** Normal **CSF:** Normal **Serum Abs:** N/A | **Local tx:** CS **IS Tx:** No **Onco Tx:** Surg | Cervical cancer,  - 7 years | Stable | Recovery |
| F. Hoogewoud, 2018, France | F, 65 | **Lat:** Bilateral **Sx:** Decreased visual acuity **VF:** Tubular **ACI:** Yes **Fundus exam:** Normal | **OCT:** Ellipsoid zone atrophy **FA:** Retinal venous vasculitis **ERG:** Pathologic (cones and rods) | **Brain MRI:** N/A **CSF:** N/A **Serum Abs:** N/A | **Local tx:** No **IS Tx:** PLEX **Onco Tx:** N/A | Uterine sarcoma, Simultaneous | Worsening | N/A |
| F. Hoogewoud, 2018, France | M, 55 | **Lat:** Bilateral **Sx:** Decreased visual acuity **VF:** Tubular **ACI:** No **Fundus exam:** Normal | **OCT:** Ellipsoid zone atrophy **FA:** Normal **ERG:** Pathologic (cones and rods) | **Brain MRI:** N/A **CSF:** N/A **Serum Abs:** N/A | **Local tx:** No **IS Tx:** IV CS **Onco Tx:** N/A | SCLC,  Simultaneous | Improvement | N/A |
| F. Hoogewoud, 2018, France | M, 75 | **Lat:** Bilateral **Sx:** Decreased visual acuity **VF:** Tubular **ACI:** No **Fundus exam:** Macular edema | **OCT:** Ellipsoid zone atrophy **FA:** Normal **ERG:** Pathologic (rods > cones) | **Brain MRI:** N/A **CSF:** N/A **Serum Abs:** N/A | **Local tx:** No **IS Tx:** CS **Onco Tx:** N/A | Prostate adenocarcinoma, Simultaneous | Worsening | N/A |
| F. Hoogewoud, 2018, France | F, 65 | **Lat:** Bilateral **Sx:** Decreased visual acuity **VF:** Tubular **ACI:** No **Fundus exam:** Normal | **OCT:** Ellipsoid zone atrophy **FA:** Normal **ERG:** Pathologic (cones and rods) | **Brain MRI:** Normal **CSF:** N/A **Serum Abs:** N/A | **Local tx:** No **IS Tx:** Azathioprine **Onco Tx:** N/A | SCLC, Simultaneous | Worsening | N/A |
| N. Ghadiri, 2019, United Kingdom | F, 64 | **Lat:** Bilateral **Sx:** Decreased visual acuity, photopsia **VF:** Paracentral scotoma **ACI:** No **Fundus exam:** Normal | **OCT:** N/A **FA:** N/A **ERG:** Pathologic (cones and rods) | **Brain MRI:** N/A **CSF:** N/A **Serum Abs:** N/A | **Local tx:** No **IS Tx:** PLEX **Onco Tx:** CT | Pancreatic adenocarcinoma, Simultaneous | Stable | Recovery |
| K. Yagyu, 2019, Japan | F, 74 | **Lat:** Bilateral **Sx:** Decreased visual acuity **VF:** Tubular **ACI:** No **Fundus exam:** Normal | **OCT:** N/A **FA:** Retinal venous vasculitis **ERG:** Pathologic (cones and rods) | **Brain MRI:** Normal **CSF:** N/A **Serum Abs:** Negative | **Local tx:** N/A **IS Tx:** IVIG, Azathioprine **Onco Tx:** RadioCT | SCLC,  Simultaneous | Improvement | Death |
| G. Merle, 2020, Switzerland | F, 77 | **Lat:** Bilateral **Sx:** Decreased visual acuity, photopsia **VF:** Diffuse defect **ACI:** No **Fundus exam:** Arterial narrowing | **OCT:** Ellipsoid zone atrophy **FA:** Retinal venous vasculitis **ERG:** Pathologic (cones and rods) | **Brain MRI:** N/A **CSF:** N/A **Serum Abs:** N/A | **Local tx:** No **IS Tx:** Cyclosporine **Onco Tx:** Surg | Bronchial carcinoid, Simultaneous | Improvement | N/A |
| N. Huynh, 2012, United States | M, 67 | **Lat:** Bilateral **Sx:** Hemeralopia **VF:** N/A **ACI:** No **Fundus exam:** Normal | **OCT:** Ellipsoid zone atrophy **FA:** Retinal venous vasculitis **ERG:** Pathologic (cones and rods) | **Brain MRI:** N/A **CSF:** N/A **Serum Abs:** Alpha enolase | **Local tx:** No **IS Tx:** CS, MMF, IVIG **Onco Tx:** CT | Squamous cell lung carcinoma, Simultaneous | Improvement | Death |
| D. Roels, 2017, Belgium | F, 45 | **Lat:** Unilateral **Sx:** Scotoma **VF:** Normal **ACI:** No **Fundus exam:** Normal | **OCT:** Normal **FA:** Normal **ERG:** Pathologic (cones and rods) | **Brain MRI:** N/A **CSF:** N/A **Serum Abs:** TRPM1 | **Local tx:** N/A **IS Tx:** CS **Onco Tx:** Surg | Ovarian adenocarcinoma, Simultaneous | Recovery | Recovery |
| M. Nivean, 2016, India | F, 71 | **Lat:** Bilateral **Sx:** Photophobia, photopsia **VF:** Severe diffuse defect **ACI:** No **Fundus exam:** Normal | **OCT:** Retinal inner layer atrophy **FA:** Normal **ERG:** Pathologic (cones and rods) | **Brain MRI:** N/A **CSF:** N/A **Serum Abs:** Negative | **Local tx:** N/A **IS Tx:** Rituximab **Onco Tx:** CT | Neuroendocrine bladder carcinoma, Simultaneous | Worsening | Death |
| Y. Sakamori, 2011, Japan | F, 65 | **Lat:** Bilateral **Sx:** Decreased visual acuity, pain, dyschromatopsia **VF:** Central scotoma **ACI:** No **Fundus exam:** Normal | **OCT:** N/A **FA:** Hemorrhage **ERG:** N/A | **Brain MRI:** N/A **CSF:** N/A **Serum Abs:** Negative | **Local tx:** N/A **IS Tx:** IV CS **Onco Tx:** RT | Metastatic SCLC, Simultaneous | Improvement | Recovery |
| Y. Sakamori, 2011, Japan | M, 88 | **Lat:** Bilateral **Sx:** Decreased visual acuity **VF:** Tubular **ACI:** No **Fundus exam:** Normal | **OCT:** N/A **FA:** N/A **ERG:** Pathologic | **Brain MRI:** Normal **CSF:** N/A **Serum Abs:** N/A | **Local tx:** N/A **IS Tx:** CS **Onco Tx:** CT | SCLC,  Simultaneous | Stable | Stable |
| W. Carrera, 2019, United States | F, 49 | **Lat:** Bilateral **Sx:** Photophobia, decreased color vision **VF:** Paracentral scotoma **ACI:** Yes **Fundus exam:** Optic disc atrophy | **OCT:** N/A **FA:** Retinal venous vasculitis **ERG:** N/A | **Brain MRI:** Normal **CSF:** Increased CSF protein **Serum Abs:** Recoverin | **Local tx:** N/A **IS Tx:** CS **Onco Tx:** CT | Metastatic lobular breast carcinoma, Simultaneous | Stable | Death |
| A. Anastasakis, 2011, United Kingdom | F, 62 | **Lat:** Bilateral **Sx:** Decreased visual acuity, photopsia **VF:** Tubular **ACI:** No **Fundus exam:** Normal | **OCT:** N/A **FA:** Retinal venous vasculitis **ERG:** Pathologic (rods) | **Brain MRI:** N/A **CSF:** N/A **Serum Abs:** N/A | **Local tx:** N/A **IS Tx:** CS **Onco Tx:** CT | SCLC,  Simultaneous | Recovery | N/A |
| H. Kitai, 2015, Japan | F, 65 | **Lat:** Bilateral **Sx:** Decreased visual acuity, photopsia **VF:** Central scotoma **ACI:** No **Fundus exam:** Normal | **OCT:** N/A **FA:** N/A **ERG:** Pathologic | **Brain MRI:** N/A **CSF:** N/A **Serum Abs:** Recoverin | **Local tx:** N/A **IS Tx:** CS **Onco Tx:** CT | SCLC,  Simultaneous | Recovery | Recovery |
| M. Morita, 2014, Japan | F, 60 | **Lat:** Bilateral **Sx:** Decreased visual acuity, photophobia **VF:** Central scotoma **ACI:** No **Fundus exam:** Normal | **OCT:** N/A **FA:** N/A **ERG:** Pathologic (cones and rods) | **Brain MRI:** N/A **CSF:** N/A **Serum Abs:** CRMP5 | **Local tx:** No **IS Tx:** No **Onco Tx:** CT | Metastatic SCLC,  +6 months | Stable | Death |
| S. Ogra, 2013, New Zealand | M, 66 | **Lat:** Bilateral **Sx:** Decreased visual acuity, dyschromatopsia **VF:** Tubular **ACI:** No **Fundus exam:** N/A | **OCT:** N/A **FA:** N/A **ERG:** Pathologic (rods > cones) | **Brain MRI:** N/A **CSF:** N/A **Serum Abs:** CA2, Alpha enolase | **Local tx:** No **IS Tx:** CS **Onco Tx:** N/A | Neuroendocrine colic carcinoma,  -22 months | N/A | Recovery |
| E. Dimitriou, 2021, Greece | F, 71 | **Lat:** Bilateral **Sx:** Hemeralopia **VF:** Tubular **ACI:** No **Fundus exam:** Vascular narrowing | **OCT:** Atrophy of outer retinal layer **FA:** Normal **ERG:** Pathologic (rods > cones) | **Brain MRI:** N/A **CSF:** N/A **Serum Abs** Alpha enolase | **Local tx:** No **IS Tx:** N/A **Onco Tx:** N/A | Ovarian carcinoma,  -4 years | N/A | N/A |
| S. Wagley, 2020, United States | F, 84 | **Lat:** Bilateral **Sx:** Hemeralopia **VF:** Tubular **ACI:** No **Fundus exam:** Normal | **OCT:** Atrophy of outer + ellipsoid retinal layers **FA:** N/A **ERG:** Pathologic (rods) | **Brain MRI:** N/A **CSF:** N/A **Serum Abs:** Recoverin, alpha enolase, GAPDH | **Local tx:** CS **IS Tx:** CS **Onco Tx:** N/A | Clear cell renal carcinoma, Simultaneous | N/A | N/A |
| S. Naramala, 2019, United States | F, 39 | **Lat:** Bilateral **Sx:** Scotoma, Hemeralopia **VF:** Central scotoma **ACI:** Yes **Fundus exam:** Retinal vasculitis | **OCT:** N/A **FA:** Hyperfluorescence **ERG:** N/A | **Brain MRI:** N/A **CSF:** N/A **Serum Abs:** Aldolase, Alpha enolase, GAPDH | **Local tx:** CS **IS Tx:** Cyclosporine **Onco Tx:** N/A | Urothelial carcinoma, Simultaneous | N/A | N/A |
| S. Naramala, 2019, United States | F, 58 | **Lat:** Bilateral **Sx:** Decreased visual acuity, color vision impairment **VF:** Tubular **ACI:** No **Fundus exam:** Papillary atrophy | **OCT:** N/A **FA:** N/A **ERG:** N/A | **Brain MRI:** Normal **CSF:** N/A **Serum Abs:** Recoverin | **Local tx:** CS **IS Tx:** MMF, IVIG **Onco Tx:** N/A | Endometrial carcinoma, Simultaneous | N/A | N/A |
| H. Imai, 2012, Japan | M, 43 | **Lat:** Unilateral **Sx:** Decreased visual acuity **VF:** Central scotoma **ACI:** No **Fundus exam:** Normal | **OCT:** Atrophy of outer retinal layer **FA:** N/A **ERG:** Pathologic (rods) | **Brain MRI:** N/A **CSF:** N/A **Serum Abs:** Negative | **Local tx:** No **IS Tx:** CS **Onco Tx:** N/A | Testicular seminoma, Simultaneous | N/A | N/A |
| T.R. Cristescu, 2016, Romania | M, 57 | **Lat:** Bilateral **Sx:** Decreased visual acuity **VF:** Central scotoma **ACI:** No **Fundus exam:** Papillary pallor | **OCT:** Macular atrophy **FA:** N/A **ERG:** N/A | **Brain MRI:** N/A **CSF:** N/A **Serum Abs:** Recoverin, alpha enolase | **Local tx:** No **IS Tx:** No **Onco Tx:** N/A | Pancreatic adenocarcinoma, Simultaneous | N/A | N/A |
| S. Ueno, 2014, Japan | F, 71 | **Lat:** Bilateral **Sx:** Photopsia **VF:** N/A **ACI:** No **Fundus exam:** Normal | **OCT:** Normal **FA:** Normal **ERG:** Pathologic (rods > cones) | **Brain MRI:** N/A **CSF:** N/A **Serum Abs:** Negative | **Local tx:** No **IS Tx:** No **Onco Tx:** Surg | Metastatic ovarian adenocarcinoma, metastatic, Simultaneous | Recovery | Recovery |
| S. Ueno, 2014, Japan | M, 69 | **Lat:** Bilateral **Sx:** Hemeralopia **VF:** N/A **ACI:** No **Fundus exam:** Choroidal hypopigmentation | **OCT:** Choroidal atrophy **FA:** N/A **ERG:** Pathologic (rods > cones) | **Brain MRI:** N/A **CSF:** N/A **Serum Abs:** TRPM1 | **Local tx:** No **IS Tx:** No **Onco Tx:** CT | SCLC,  Simultaneous | Stable | Recovery |
| Y. Liu, 2014, United States | M, 58 | **Lat:** Bilateral **Sx:** Decreased visual acuity **VF:** N/A **ACI:** No **Fundus exam:** Normal | **OCT:** N/A **FA:** Normal **ERG:** Pathologic (rods) | **Brain MRI:** N/A **CSF:** N/A **Serum Abs:** GAPDH | **Local tx:** No **IS Tx:** Rituximab **Onco Tx:** CT | Waldenström’s macroglobulinemia,  -7 years | Stable | Recovery |
| R. Myadham, 2020, United States | M, 35 | **Lat:** Bilateral **Sx:** Phosphene scotoma **VF:** Tubular **ACI:** Yes **Fundus exam:** Normal | **OCT:** N/A **FA:** Vasculitis **ERG:** N/A | **Brain MRI:** Normal **CSF:** Normal **Serum Abs:** Recoverin, CA2, Aldolase, Alpha enolase, GAPDH | **Local tx:** No **IS Tx:** PLEX **Onco Tx:** RT | Metastatic testicular seminoma, Simultaneous | Worsening | Death |
| M. Kondo, 2010, Japan | M, 42 | **Lat:** Bilateral **Sx:** Decreased visual acuity, dyschromatopsia **VF:** Central scotoma **ACI:** No **Fundus exam:** Optic disc edema | **OCT:** Serous retinal detachment **FA:** Vasculitis **ERG:** Pathologic (cones and rods) | **Brain MRI:** N/A **CSF:** N/A **Serum Abs:** Recoverin | **Local tx:** No **IS Tx:** PLEX **Onco Tx:** CT | Retroperitoneal liposarcoma, Simultaneous | N/A | N/A |
| M. Saito, 2014, Japan | M, 67 | **Lat:** Bilateral **Sx:** Decreased visual acuity, color vision deficit **VF:** Central scotoma **ACI:** Yes **Fundus exam:** Venous dilation | **OCT:** N/A **FA:** N/A **ERG:** N/A | **Brain MRI:** Normal **CSF:** N/A **Serum Abs:** Recoverin, CRMP5, Alpha enolase | **Local tx:** No **IS Tx:** No **Onco Tx:** Surg | SCLC,  Simultaneous | Recovery | Recovery |
| A. Raghunath, 2010, United States | F, 70 | **Lat:** Bilateral **Sx:** Decreased visual acuity, dyschromatopsia **VF:** Paracentral scotoma **ACI:** Yes **Fundus exam:** Hyalitis | **OCT:** Macular edema **FA:** Vasculitis **ERG:** Pathologic (cones and rods) | **Brain MRI:** White matter anomaly **CSF:** Normal **Serum Abs:** CA2, Alpha enolase | **Local tx:** No **IS Tx:** No **Onco Tx:** CT | Neuroendocrine carcinoma of Fallopian tube, Simultaneous | Stable | N/A |
| T. Nakamura, 2015, Japan | M, 59 | **Lat:** Bilateral **Sx:** Hemeralopia **VF:** Tubular **ACI:** No **Fundus exam:** Arterial narrowing | **OCT:** N/A **FA:** N/A **ERG:** Pathologic (rods) | **Brain MRI:** N/A **CSF:** N/A **Serum Abs:** N/A | **Local tx:** No **IS Tx:** No **Onco Tx:** Surg | Large-cell pulmonary neuroendocrine carcinoma, Simultaneous | Improvement | Recovery |
| K. Mizobuchi, 2020, Japan | F, 67 | **Lat:** Bilateral **Sx:** Photophobia **VF:** Paracentral scotoma **ACI:** No **Fundus exam:** Normal | **OCT:** Outer + ellipsoid layer atrophy **FA:** Retinal venous vasculitis **ERG:** Pathologic (cones and rods) | **Brain MRI:** N/A **CSF:** N/A **Serum Abs:** N/A | **Local tx:** CS **IS Tx:** CS **Onco Tx:** Surg | Uterine carcinoma,  -12 months | Worsening | Progression |
| A. Andrikopoulou, 2023, Greece | F, 67 | **Lat:** Unilateral **Sx:** Color vision deficit **VF:** Tubular **ACI:** No **Fundus exam:** Arterial narrowing | **OCT:** Outer + ellipsoid layer atrophy **FA:** Normal **ERG:** Pathologic (rods > cones) | **Brain MRI:** N/A **CSF:** N/A **Serum Abs:** Alpha enolase | **Local tx:** IVIG **IS Tx:** Rituximab **Onco Tx:** CT | Ovarian carcinoma, Simultaneous | Improvement | Recovery |
| M. Thomas, 2023, United States | F, 66 | **Lat:** Bilateral **Sx:** Decreased visual acuity **VF:** Central scotoma **ACI:** No **Fundus exam:** N/A | **OCT:** Normal **FA:** N/A **ERG:** N/A | **Brain MRI:** Normal **CSF:** N/A **Serum Abs:** Negative | **Local tx:** PLEX **IS Tx:** Cyclophosphamide **Onco Tx:** Surg | Bronchial small cell carcinoma, Simultaneous | Worsening | Progression |
| B. Weixler, 2016, Switzerland | M, 76 | **Lat:** Bilateral **Sx:** Myodesopsia, phosphenes **VF:** Central scotoma **ACI:** No **Fundus exam:** Arterial narrowing | **OCT:** N/A **FA:** N/A **ERG:** Pathologic (cones and rods) | **Brain MRI:** Normal **CSF:** N/A **Serum Abs:** Negative | **Local tx:** CS **IS Tx:** IVIG **Onco Tx:** RT | Colic adenocarcinoma, Simultaneous | Worsening | Death |
| A. Sampedro, 2013, Spain | F, 72 | **Lat:** Bilateral **Sx:** Decreased visual acuity **VF:** N/A **ACI:** No **Fundus exam:** N/A | **OCT:** N/A **FA:** Vasculitis **ERG:** Pathologic (cones and rods) | **Brain MRI:** N/A **CSF:** N/A **Serum Abs:** N/A | **Local tx:** No **IS Tx:** No **Onco Tx:** CT | Undifferentiated pelvic neoplasia,  -6 months | Worsening | N/A |
| S.J. Kim, 2010, United States | F, 60 | **Lat:** Bilateral **Sx:** Decreased visual acuity **VF:** Central scotoma **ACI:** No **Fundus exam:** Hyalitis | **OCT:** N/A **FA:** Choroidal hyperfluorescence **ERG:** Pathologic | **Brain MRI:** N/A **CSF:** N/A **Serum Abs:** Recoverin | **Local tx:** No **IS Tx:** CS **Onco Tx:** CT | Metastatic ovarian carcinoma, Simultaneous | Stable | Death |
| I. Dy, 2013, United States | F, 61 | **Lat:** Bilateral **Sx:** Myodesopsia, photopsia **VF:** Tubular **ACI:** No **Fundus exam:** Periphlebitis | **OCT:** N/A **FA:** Choroidal hyperfluorescence **ERG:** Pathologic (cones and rods) | **Brain MRI:** N/A **CSF:** N/A **Serum Abs:** Negative | **Local tx:** No **IS Tx:** CS **Onco Tx:** Surg | Neuroendocrine small cell uterine carcinoma, Simultaneous | Improvement | Death |
| J.J. Lee, 2015, United States | F, 54 | **Lat:** Bilateral **Sx:** Decreased visual acuity **VF:** N/A **ACI:** No **Fundus exam:** Normal | **OCT:** N/A **FA:** Choroidal hypofluorescence **ERG:** N/A | **Brain MRI:** N/A **CSF:** N/A **Serum Abs:** Recoverin | **Local tx:** No **IS Tx:** IVIG **Onco Tx:** CT | Neuroendocrine small cell uterine carcinoma,  +2 months | Worsening | Death |
| C. Duncan, 2019, Australia | M, 71 | **Lat:** Bilateral **Sx:** Hemeralopia **VF:** Tubular **ACI:** Yes **Fundus exam:** Hyalitis | **OCT:** N/A **FA:** N/A **ERG:** Pathologic (cones and rods) | **Brain MRI:** Normal **CSF:** N/A **Serum Abs:** N/A | **Local tx:** No **IS Tx:** Rituximab **Onco Tx:** N/A | Small cell prostate carcinoma, Simultaneous | Improvement | Improvement |
| M. Kamei, 2018, Japan | F, 50 | **Lat:** Bilateral **Sx:** Photopsia **VF:** Paracentral scotoma **ACI:** No **Fundus exam:** Retinal lesions | **OCT:** Photoreceptor atrophy **FA:** Choroidal hyperfluorescence **ERG:** Pathologic | **Brain MRI:** Normal **CSF:** N/A **Serum Abs:** Recoverin | **Local tx:** No **IS Tx:** MMF **Onco Tx:** CT | Breast carcinoma with neuroendocrine component,  -1 month | Worsening | Improvement |
| P. Cybulska, 2011, Canada | F, 62 | **Lat:** Bilateral **Sx:** Scintillating scotoma **VF:** N/A **ACI:** No **Fundus exam:** Hyalitis | **OCT:** Serous retinal detachment **FA:** Choroidal hyperfluorescence **ERG:** N/A | **Brain MRI:** N/A **CSF:** N/A **Serum Abs:** N/A | **Local tx:** No **IS Tx:** CS **Onco Tx:** Surg | Clear cell uterine carcinoma,  -1 month | Improvement | Improvement |
| D. Chao, 2013, United States | F, 62 | **Lat:** Bilateral **Sx:** Decreased visual acuity **VF:** Tubular **ACI:** No **Fundus exam:** Normal | **OCT:** Global retinal atrophy **FA:** Choroidal hyperfluorescence **ERG:** Pathologic (cones and rods) | **Brain MRI:** Normal **CSF:** N/A **Serum Abs:** Alpha enolase, alpha transducin | **Local tx:** No **IS Tx:** PLEX **Onco Tx:** CT | Colic adenocarcinoma, Simultaneous | Improvement | RT |
| C. Martinez, 2020, Spain | M, 59 | **Lat:** Bilateral **Sx:** Decreased visual acuity **VF:** Severe alteration **ACI:** No **Fundus exam:** Papilledema | **OCT:** Outer + ellipsoid layer atrophy **FA:** Venous vasculitis **ERG:** Pathologic (cones and rods) | **Brain MRI:** Normal **CSF:** N/A **Serum Abs:** CRMP5 | **Local tx:** No **IS Tx:** CS **Onco Tx:** CT | SCLC,  Simultaneous | Worsening | N/A |
| A. Ghosh, 2022, United States | M, 72 | **Lat:** Bilateral **Sx:** Decreased visual acuity **VF:** Central scotoma **ACI:** No **Fundus exam:** Hyalitis | **OCT:** Global retinal atrophy **FA:** N/A **ERG:** Pathologic | **Brain MRI:** N/A **CSF:** N/A **Serum Abs:** Positive (unspecified) | **Local tx:** No **IS Tx:** No **Onco Tx:** Surg | Renal carcinoma, Simultaneous | Worsening | Progression |
| J. Eadie, 2014, United States | F, 67 | **Lat:** Bilateral **Sx:** Scotoma **VF:** N/A **ACI:** No **Fundus exam:** Normal | **OCT:** Inner retinal atrophy **FA:** Diffuse hyperfluorescence **ERG:** Pathologic | **Brain MRI:** N/A **CSF:** N/A **Serum Abs:** HSP | **Local tx:** No **IS Tx:** No **Onco Tx:** Immunotherapy | Breast cancer,  -60 months | Worsening | Recovery |
| G. de Saint Sauveur, 2021, France | M, 56 | **Lat:** Bilateral **Sx:** Photopsia **VF:** Severe alteration **ACI:** No **Fundus exam:** Normal | **OCT:** Paracentral scotoma **FA:** Paravascular hyperfluorescence **ERG:** Pathologic | **Brain MRI:** Normal **CSF:** N/A **Serum Abs:** Negative | **Local tx:** No **IS Tx:** PLEX **Onco Tx:** Surg | Metastatic SCLC,  -5 months | Worsening | Death |
| F.K. Chen, 2017, Australia | M, 59 | **Lat:** Bilateral **Sx:** Decreased visual acuity **VF:** Central scotoma **ACI:** No **Fundus exam:** Normal | **OCT:** Ellipsoid layer atrophy **FA:** Normal **ERG:** Pathologic (cones) | **Brain MRI:** Normal **CSF:** N/A **Serum Abs:** Alpha enolase, GAPDH | **Local tx:** No **IS Tx:** IVIG **Onco Tx:** RT | Neuroendocrine prostate tumor,  +6 months | Improvement | N/A |
| E. Hughes, 2016, Ireland | M, 48 | **Lat:** Bilateral **Sx:** Decreased visual acuity **VF:** Paracentral scotoma **ACI:** No **Fundus exam:** Normal | **OCT:** Normal **FA:** N/A **ERG:** Pathologic (cones and rods) | **Brain MRI:** N/A **CSF:** N/A **Serum Abs:** N/A | **Local tx:** Intravitreal CS **IS Tx:** CS **Onco Tx:** CT | Papillary thyroid carcinoma, Simultaneous | Improvement | Recovery |
| W. Saito, 2013, Japan | M, 73 | **Lat:** Bilateral **Sx:** Hemeralopia **VF:** Central scotoma **ACI:** No **Fundus exam:** Optic atrophy | **OCT:** Outer retinal atrophy **FA:** Diffuse hyperfluorescence **ERG:** Pathologic (cones and rods) | **Brain MRI:** N/A **CSF:** N/A **Serum Abs:** Recoverin | **Local tx:** No **IS Tx:** CS, Azathioprine **Onco Tx:** Surg | Colic adenocarcinoma,  +24 months | Improvement | Stable |
| L. Moyal, 2017, France | M, 70 | **Lat:** Bilateral **Sx:** Photophobia **VF:** N/A **ACI:** No **Fundus exam:** Hyalitis | **OCT:** Ellipsoid layer atrophy **FA:** Normal **ERG:** Pathologic (cones and rods) | **Brain MRI:** N/A **CSF:** N/A **Serum Abs:** Positive (unspecified) | **Local tx:** No **IS Tx:** No **Onco Tx:** Surg | Thymoma, Simultaneous | Worsening | N/A |
| P. Sanghi, 2021, United Kingdom | F, 41 | **Lat:** Bilateral **Sx:** Color vision alteration **VF:** Paracentral scotoma **ACI:** No **Fundus exam:** Normal | **OCT:** Outer retinal atrophy **FA:** Normal **ERG:** Pathologic (cones > rods) | **Brain MRI:** Normal **CSF:** N/A **Serum Abs:** Negative | **Local tx:** No **IS Tx:** No **Onco Tx:** CT | DLBCL,  +12 months | Stable | Recovery |

**ACI:** Anterior chamber inflammation, **Serum Abs:** Antibody, **CA:** Carbonic anhydrase II, **CRMP5:** Collapsin response mediator protein 5, **CS:** Corticosteroids, **CSF:** Cerebrospinal fluid, **CT:** Chemotherapy, **ERG:** Electroretinogram, **FA:** Fluorescein angiography, **GAPDH:** Glyceraldehyde 3-phosphate dehydrogenase, **HSP:** Heat shock protein, **IS Tx:** Immunosuppressive treatment, **IV:** Intravenous, **IV CS:** Intravenous corticosteroids, **IVIG:** Intravenous immunoglobulin, **DLBCL:** Diffuse large B-cell lymphoma, **Lat:** Laterality, **MMF:** Mycophenolate mofetil, **MRI:** Magnetic resonance imaging, **N/A:** Not available, **OCT:** Optical coherence tomography, **Onco Tx:** Oncological treatment, **PLEX:** Plasma exchange, **RT:** radiotherapy, **SCLC:** Small cell lung cancer, **Surg**: Surgery, **Sx:** Symptoms, **TRPM1:** Transient receptor potential cation channel subfamily M member 1, **Tx:** Treatment, **VF:** Visual field.
