# Supplementary material for: Paraneoplastic ocular syndromes: a systematic review of epidemiology, diagnosis and outcomes (2010–2023)
Source: J Ophthalmic Inflamm Infect. 2025 Sep 26;15:73. doi: 10.1186/s12348-025-00534-1 (PMC12474834; doi:10.1186/s12348-025-00534-1)
Supplement: Supplementary file 2 — Supplementary Material 2. [file 12348_2025_534_MOESM2_ESM.docx]

**Table 6: Characteristics of reported cases of melanoma associated retinopathy.**

| **Author, year, country** | **Sex, age** | **Ophthalmologic data** | **Ophthalmologic exams** | **Systemic workup** | **Treatment** | **Cancer, diagnosis timing** | **Visual outcome** | **Cancer outcome** |
| --- | --- | --- | --- | --- | --- | --- | --- | --- |
| H. Bahig, Canada, 2011 | F, 67 | **Laterality:** Bilateral **Symptoms:** Visual loss **AC inflammation:** N/A **Fundus exam:** N/A | **OCT:** N/A **FA:** N/A **ERG:** Abnormal (rods) | **Brain MRI:** Normal **CSF analysis:** N/A **Serum Abs:** N/A | **Local tx:** No **IS Tx:** No **Onco Tx:** No | Metastatic melanoma -108 months | Recovery | Recovery |
| M. Heberton, USA, 2019 | M, 74 | **Laterality:** Bilateral **Symptoms:** Hemeralopia **AC inflammation:** No **Fundus exam:** Normal | **OCT:** N/A **FA:** N/A **ERG:** Abnormal (rods) | **Brain MRI:** Normal **CSF analysis:** N/A **Serum Abs:** Positive (unspecified) | **Local tx:** N/A **IS Tx:** No **Onco Tx:** N/A | Metastatic melanoma recurrence -4 months | N/A | N/A |
| A. Audemard, France, 2013 | F, 70 | **Laterality:** Unilateral **Symptoms:** Photopsia **AC inflammation:** No **Fundus exam:** Vascular irregularity | **OCT:** N/A **FA:** Vasculitis **ERG:** Abnormal (rods) | **Brain MRI:** Normal **CSF analysis:** N/A **Serum Abs:** N/A | **Local tx:** No **IS Tx:** Corticosteroids **Onco Tx:** Surgery | Metastatic melanoma recurrence Simultaneous recurrence | Worsening | Stable |
| MS. Kim, South Korea, 2020 | M, 58 | **Laterality:** Bilateral **Symptoms:** Photopsia **AC inflammation:** No **Fundus exam:** Vitritis | **OCT:** Normal **FA:** Choroidal neovascularization **ERG:** Abnormal (rods) | **Brain MRI:** Normal **CSF analysis:** N/A **Serum Abs:** TRPM1 | **Local tx:** No **IS Tx:** Corticosteroids **Onco Tx:** Chemotherapy | Metastatic melanoma -36 months | Worsening | N/A |
| A. Abou-Samra, USA, 2021 | M, 63 | **Laterality:** Bilateral **Symptoms:** Hemeralopia **AC inflammation:** No **Fundus exam:** Choroidal lesion | **OCT:** Retinal atrophy **FA:** Vasculitis **ERG:** Abnormal (cones and rods) | **Brain MRI:** Normal **CSF analysis:** N/A **Serum Abs:** Aldolase C | **Local tx:** Intravitreal corticosteroid implant **IS Tx:** No **Onco Tx:** Immunotherapy | Metastatic melanoma Simultaneous recurrence | Improvement | N/A |
| S. Machida, Japan, 2011 | M, 77 | **Laterality:** Bilateral **Symptoms:** Visual loss **AC inflammation:** No **Fundus exam:** Vitritis | **OCT:** Retinal atrophy **FA:** N/A **ERG:** Abnormal (cones > rods) | **Brain MRI:** Normal **CSF analysis:** N/A **Serum Abs:** N/A | **Local tx:** No **IS Tx:** No **Onco Tx:** Surgery | Melanoma Simultaneous | Worsening | Recovery |
| P. Peeters, Belgium, 2023 | F, 72 | **Laterality:** Bilateral **Symptoms:** Visual loss **AC inflammation:** No **Fundus exam:** Vitreous detachment | **OCT:** Ellipsoid and interdigitation zone loss **FA:** Normal **ERG:** Abnormal (cones > rods) | **Brain MRI:** Normal **CSF analysis:** N/A **Serum Abs:** Negative | **Local tx:** No **IS Tx:** IV corticosteroids **Onco Tx:** Radiotherapy | Melanoma +24 months | Improvement | N/A |
| JJ. Keppi, France, 2016 | F, 83 | **Laterality:** Bilateral **Symptoms:** Visual loss **AC inflammation:** No **Fundus exam:** Optic disc pallor | **OCT:** Normal **FA:** N/A **ERG:** Abnormal (cones and rods) | **Brain MRI:** N/A **CSF analysis:** N/A **Serum Abs:** Anti-bipolar cells | **Local tx:** No **IS Tx:** Corticosteroids **Onco Tx:** Surgery | Melanoma recurrence Simultaneous | N/A | N/A |
| C. Ng, USA, 2023 | M, 68 | **Laterality:** Bilateral **Symptoms:** Visual loss **AC inflammation:** No **Fundus exam:** Normal | **OCT:** N/A **FA:** Diffuse hyperfluorescence **ERG:** Abnormal (rods) | **Brain MRI:** N/A **CSF analysis:** N/A **Serum Abs:** Enolase | **Local tx:** No **IS Tx:** No **Onco Tx:** Surgery | Melanoma recurrence -12 months | Improvement | Recovery |
| K. Khaddour, USA, 2020 | M, 74 | **Laterality:** Bilateral **Symptoms:** Hemeralopia **AC inflammation:** No **Fundus exam:** Normal | **OCT:** Normal **FA:** N/A **ERG:** Abnormal (rods) | **Brain MRI:** N/A **CSF analysis:** N/A **Serum Abs:** Arrestin | **Local tx:** No **IS Tx:** No **Onco Tx:** Surgery | Metastatic melanoma recurrence Simultaneous | Recovery | Recovery |
| M. Handler, USA, 2011 | M, 57 | **Laterality:** Bilateral **Symptoms:** Visual loss **AC inflammation:** No **Fundus exam:** Normal | **OCT:** N/A **FA:** N/A **ERG:** Abnormal (cones and rods) | **Brain MRI:** N/A **CSF analysis:** N/A **Serum Abs:** Pyruvate kinase M2 | **Local tx:** Corticosteroids **IS Tx:** No **Onco Tx:** Immunotherapy | Metastatic melanoma recurrence Simultaneous | Improvement | N/A |
| R. Stead, UK, 2013 | F, 72 | **Laterality:** Bilateral **Symptoms:** Hemeralopia **AC inflammation:** No **Fundus exam:** Normal | **OCT:** N/A **FA:** N/A **ERG:** Abnormal (rods) | **Brain MRI:** N/A **CSF analysis:** N/A **Serum Abs:** Negative | **Local tx:** No **IS Tx:** No **Onco Tx:** Targeted therapy | Melanoma +19 months | Recovery | Death |
| Y. Shinohara, Japan, 2021 | M, 74 | **Laterality:** Bilateral **Symptoms:** Photopsia **AC inflammation:** No **Fundus exam:** Normal | **OCT:** Interdigitation zone loss **FA:** N/A **ERG:** Abnormal (rods > cones) | **Brain MRI:** N/A **CSF analysis:** N/A **Serum Abs:** Positive | **Local tx:** No **IS Tx:** No **Onco Tx:** Surgery | Metastatic melanoma -24 months | Stable | N/A |
| A. Vercio, USA, 2021 | F, 63 | **Laterality:** Bilateral **Symptoms:** Visual loss **AC inflammation:** No **Fundus exam:** Vascular tortuosity | **OCT:** Optic nerve fiber layer loss **FA:** N/A **ERG:** Abnormal (rods) | **Brain MRI:** N/A **CSF analysis:** N/A **Serum Abs:** Ma/TRPM1 | **Local tx:** No **IS Tx:** No **Onco Tx:** Immunotherapy | Metastatic melanoma +2 months | Improvement | Improvement |
| S. Ueno, Japan, 2014 | M, 61 | **Laterality:** Bilateral **Symptoms:** Hemeralopia **AC inflammation:** No **Fundus exam:** Normal | **OCT:** Normal **FA:** Normal **ERG:** Abnormal (cones and rods) | **Brain MRI:** N/A **CSF analysis:** N/A **Serum Abs:** TRPM1 | **Local tx:** No **IS Tx:** N/A **Onco Tx:** N/A | Melanoma -4 months | Improvement | N/A |
| P. Roberts, USA, 2016 | M, N/A | **Laterality:** Bilateral **Symptoms:** VF impairment **AC inflammation:** No **Fundus exam:** Vitritis | **OCT:** Normal **FA:** Diffuse hyperfluorescence **ERG:** Abnormal (rods) | **Brain MRI:** N/A **CSF analysis:** N/A **Serum Abs:** CA2 | **Local tx:** No **IS Tx:** Corticosteroids **Onco Tx:** Surgery | Metastatic melanoma -19 months | Stable | N/A |
| A. Patel, UK, 2015 | M, 59 | **Laterality:** Bilateral **Symptoms:** Blurred vision **AC inflammation:** N/A **Fundus exam:** N/A | **OCT:** N/A **FA:** N/A **ERG:** Abnormal (rods) | **Brain MRI:** Normal **CSF analysis:** N/A **Serum Abs:** Enolase | **Local tx:** No **IS Tx:** N/A **Onco Tx:** Immunotherapy | Melanoma Simultaneous | Improvement | N/A |
| Y. Mojita, Japan, 2013 | M, 82 | **Laterality:** Bilateral **Symptoms:** Hemeralopia **AC inflammation:** N/A **Fundus exam:** N/A | **OCT:** Normal **FA:** N/A **ERG:** Abnormal (rods) | **Brain MRI:** Normal **CSF analysis:** N/A **Serum Abs:** Aldolase | **Local tx:** No **IS Tx:** Corticosteroids **Onco Tx:** Surgery | Metastatic recurrence Simultaneous | Improvement | Death |
| D. Dalal, USA, 2013 | M, 60 | **Laterality:** Bilateral **Symptoms:** Floaters **AC inflammation:** No **Fundus exam:** Vitritis | **OCT:** Choroidal neovascularization **FA:** Choroidal neovascularization **ERG:** Abnormal (rods) | **Brain MRI:** Normal **CSF analysis:** N/A **Serum Abs:** Positive (unspecified) | **Local tx:** Corticosteroids **IS Tx:** IVIG **Onco Tx:** Chemotherapy | Metastatic melanoma Simultaneous | Worsening | N/A |
| D. Rappoport, Israel, 2012 | M, 55 | **Laterality:** Bilateral **Symptoms:** Photopsia **AC inflammation:** No **Fundus exam:** Retinal hemorrhage | **OCT:** N/A **FA:** Vasculitis **ERG:** Abnormal (rods > cones) | **Brain MRI:** Normal **CSF analysis:** N/A **Serum Abs:** TRPM1 | **Local tx:** No **IS Tx:** No **Onco Tx:** Surgery | Metastatic melanoma +4 months | Improvement | Death |
| E. Karatsai, UK, 2019 | F, 73 | **Laterality:** Bilateral **Symptoms:** VF impairment **AC inflammation:** No **Fundus exam:** Vascular narrowing | **OCT:** Normal **FA:** Normal **ERG:** Abnormal (cones and rods) | **Brain MRI:** N/A **CSF analysis:** N/A **Serum Abs:** TRPM1 | **Local tx:** Corticosteroid implant **IS Tx:** No **Onco Tx:** Surgery | Metastatic melanoma -24 months | Improvement | Recovery |
| N. Richarz, Spain, 2019 | M, 48 | **Laterality:** Bilateral **Symptoms:** Visual loss **AC inflammation:** N/A **Fundus exam:** Normal | **OCT:** Normal **FA:** N/A **ERG:** Abnormal (rods) | **Brain MRI:** Normal **CSF analysis:** N/A **Serum Abs:** N/A | **Local tx:** No **IS Tx:** No **Onco Tx:** Chemotherapy, Radiotherapy, Targeted therapy | Metastatic melanoma Simultaneous | Recovery | Recovery |

**Ab:** Antibody, **AC inflammation:** Anterior chamber inflammation, **CSF:** Cerebrospinal fluid, **ERG:** Electroretinogram, **FA:** Fluorescein angiography, **Fundus exam:** Fundus examination, **IS Tx:** Immunosuppressive treatment, **IVIG:** Intravenous immunoglobulins, **Local tx:** Local treatment, **MRI:** Magnetic resonance imaging, **N/A:** Not available, **OCT:** Optical coherence tomography, **Onco Tx:** Oncological treatment, **TRPM1:** Transient Receptor Potential Cation Channel Subfamily M Member 1, **VF impairment:** Visual field impairment.
