# Supplementary material for: Paraneoplastic ocular syndromes: a systematic review of epidemiology, diagnosis and outcomes (2010–2023)
Source: J Ophthalmic Inflamm Infect. 2025 Sep 26;15:73. doi: 10.1186/s12348-025-00534-1 (PMC12474834; doi:10.1186/s12348-025-00534-1)
Supplement: Supplementary file 3 — Supplementary Material 3. [file 12348_2025_534_MOESM3_ESM.docx]

**Table 7: Characteristics of reported cases of BDUMP**

| **Author, year, country** | **Sex, age** | **Ophthalmologic data** | **Ophthalmologic exams** | **Systemic workup** | **Treatment** | **Cancer, diagnosis timing** | **Visual outcome** | **Cancer outcome** |
| --- | --- | --- | --- | --- | --- | --- | --- | --- |
| G. Allon, 2023, France | M, 71 | **Laterality:** Bilateral **Symptoms:** ↓ visual acuity **AC inflammation:** No **Fundus exam:** Exud. RD | **OCT:** RPE atrophy-thickening alternation **FA:** N/A **ERG:** Pathologic | **Brain MRI:** Normal **CSF:** N/A **Serum Abs:** N/A | **Local Tx:** No **IS Tx:** No **Onco Tx:** RadioTx | Prostate ADK Simultaneous | Improvement | N/A |
| T. Ando, 2022, Japan | F, 55 | **Laterality:** Bilateral **Symptoms:** ↓ visual acuity **AC inflammation:** No **Fundus exam:** Pigmt. lesions | **OCT:** Exud. RD **FA:** Hyperfluorescent lesions **ERG:** N/A | **Brain MRI:** N/A **CSF:** N/A **Serum Abs:** N/A | **Local Tx:** Steroids **IS Tx:** No **Onco Tx:** H. therapy | Ovarian ADK Simultaneous | Stable | Death |
| F. Antaki, 2022, Canada | M, 54 | **Laterality:** Bilateral **Symptoms:** ↓ visual acuity **AC inflammation:** No **Fundus exam:** Pigmt. chor. lesions | **OCT:** RPE thickening and detach. **FA:** Hyperfluorescent lesions **ERG:** N/A | **Brain MRI:** N/A **CSF:** N/A **Serum Abs:** N/A | **Local Tx:** No **IS Tx:** Plasmapheresis **Onco Tx:** Surgery | Renal CCC Simultaneous | Recovery | Improvement |
| G. Ayachit, 2018, India | F, 67 | **Laterality:** Unilateral **Symptoms:** ↓ visual acuity **AC inflammation:** No **Fundus exam:** Exud. RD | **OCT:** Serous RD **FA:** Hyperfluorescent lesions **ERG:** N/A | **Brain MRI:** N/A **CSF:** N/A **Serum Abs:** N/A | **Local Tx:** Beva. **IS Tx:** No **Onco Tx:** ChemoTx | Met. breast Ca Simultaneous | Improvement | N/A |
| M. Boukari, 2021, Tunisia | M, 75 | **Laterality:** Bilateral **Symptoms:** Metamorphopsia **AC inflammation:** No **Fundus exam:** Pigmt. chor. lesions | **OCT:** Serous exudative RD **FA:** Hyperfluorescent lesions **ERG:** N/A | **Brain MRI:** N/A **CSF:** N/A **Serum Abs:** N/A | **Local Tx:** No **IS Tx:** No **Onco Tx:** ImmunoTx | Lung ADK Anterior | N/A | N/A |
| N. Draca, 2023, Croatia | F, 62 | **Laterality:** Bilateral **Symptoms:** ↓ visual acuity **AC inflammation:** Yes **Fundus exam:** Pigmt. chor. lesions | **OCT:** RPE irregularity **FA:** N/A **ERG:** N/A | **Brain MRI:** N/A **CSF:** N/A **Serum Abs:** N/A | **Local Tx:** Steroids **IS Tx:** Plasmapheresis **Onco Tx:** ChemoTx | Clear cell endom. Ca - 9 months | Worsening | N/A |
| S. Inoda, 2022, Japan | M, 72 | **Laterality:** Bilateral **Symptoms:** ↓ visual acuity **AC inflammation:** No **Fundus exam:** Pigmt. chor. lesions | **OCT:** Exud. RD **FA:** Hyperfluorescent lesions **ERG:** N/A | **Brain MRI:** N/A **CSF:** N/A **Serum Abs:** N/A | **Local Tx:** No **IS Tx:** Steroids **Onco Tx:** ImmunoTx | SCLC - 5 months | Stable | Worsening |
| G. Jansen, 2015, Belgium | M, 66 | **Laterality:** Bilateral **Symptoms:** ↓ visual acuity **AC inflammation:** No **Fundus exam:** Pigmt. retinal lesions | **OCT:** Exud. RD **FA:** Hyperfluorescent lesions **ERG:** N/A | **Brain MRI:** N/A **CSF:** N/A **Serum Abs:** N/A | **Local Tx:** Beva. **IS Tx:** No **Onco Tx:** RadioTx | Lung ADK Simultaneous | Improvement | Improvement |
| H. Zemirli, 2023, France | M, 70 | **Laterality:** Bilateral **Symptoms:** ↓ visual acuity **AC inflammation:** No **Fundus exam:** Pigmt. retinal lesions | **OCT:** Serous RD **FA:** Hyperfluorescent lesions **ERG:** N/A | **Brain MRI:** N/A **CSF:** N/A **Serum Abs:** N/A | **Local Tx:** No **IS Tx:** Plasmapheresis **Onco Tx:** ChemoTx | Metastatic clear cell renal carcinoma Anterior | Improvement | Stable |
| G. Jansen, 2015, Belgium | M, 67 | **Laterality:** Bilateral **Symptoms:** ↓ visual acuity **AC inflammation:** No **Fundus exam:** Pigmt. retinal lesions | **OCT:** RPE atrophy-thickening alternation **FA:** Hyperfluorescent lesions **ERG:** N/A | **Brain MRI:** N/A **CSF:** N/A **Serum Abs:** N/A | **Local Tx:** No **IS Tx:** Plasmapheresis **Onco Tx:** RadioTx | SCLC Simultaneous | Stable | Death |
| E. Katzburg, 2021, Israel | F, 61 | **Laterality:** Bilateral **Symptoms:** ↓ visual acuity **AC inflammation:** No **Fundus exam:** Pigmt. retinal lesions | **OCT:** Exud. RD **FA:** Hyperfluorescent lesions **ERG:** N/A | **Brain MRI:** N/A **CSF:** N/A **Serum Abs:** N/A | **Local Tx:** No **IS Tx:** N/A **Onco Tx:** ChemoTx | Met. SCC - 15 months | Recovery | Recovery |
| M. Luo, 2020, China | M, 50 | **Laterality:** Bilateral **Symptoms:** ↓ visual acuity **AC inflammation:** No **Fundus exam:** Pigmt. retinal lesions | **OCT:** RPE atrophy-thickening alternation **FA:** Hyperfluorescent lesions **ERG:** N/A | **Brain MRI:** N/A **CSF:** N/A **Serum Abs:** N/A | **Local Tx:** No **IS Tx:** Plasmapheresis **Onco Tx:** Surgery | Gastric ADK Simultaneous | N/A | N/A |
| C. Menezes, 2016, Portugal | M, 67 | **Laterality:** Unilateral **Symptoms:** ↓ visual acuity **AC inflammation:** No **Fundus exam:** Pigmt. retinal lesions | **OCT:** Exud. RD **FA:** Hyperfluorescent lesions **ERG:** N/A | **Brain MRI:** N/A **CSF:** N/A **Serum Abs:** N/A | **Local Tx:** No **IS Tx:** No **Onco Tx:** ChemoTx | Met. lung ADK Simultaneous | Recovery | Worsening |
| RB. Mets, 2011, USA | M, 72 | **Laterality:** Bilateral **Symptoms:** ↓ visual acuity **AC inflammation:** No **Fundus exam:** Exud. RD | **OCT:** Exud. RD **FA:** Hyperfluorescent lesions **ERG:** N/A | **Brain MRI:** N/A **CSF:** N/A **Serum Abs:** N/A | **Local Tx:** No **IS Tx:** No **Onco Tx:** N/A | SCLC - 4 months | Worsening | Worsening |
| S. Parakh, 2022, India | M, 58 | **Laterality:** Bilateral **Symptoms:** ↓ visual acuity **AC inflammation:** No **Fundus exam:** Pigmt. retinal lesions | **OCT:** RPE atrophy-thickening alternation **FA:** Hyperfluorescent lesions **ERG:** N/A | **Brain MRI:** N/A **CSF:** N/A **Serum Abs:** N/A | **Local Tx:** No **IS Tx:** Plasmapheresis **Onco Tx:** ChemoTx | Renal CCC Simultaneous | Worsening | N/A |
| M. Prasuhn, 2021, Germany | M, 76 | **Laterality:** Bilateral **Symptoms:** ↓ visual acuity **AC inflammation:** No **Fundus exam:** Pigmt. retinal lesions | **OCT:** Exud. RD **FA:** N/A **ERG:** N/A | **Brain MRI:** N/A **CSF:** N/A **Serum Abs:** N/A | **Local Tx:** Ranibizumab **IS Tx:** Steroids **Onco Tx:** ChemoTx | SCLC + 2 months | N/A | N/A |
| V. Raval, 2019, India | M, 79 | **Laterality:** Bilateral **Symptoms:** ↓ visual acuity **AC inflammation:** No **Fundus exam:** Pigmt. retinal lesions | **OCT:** Exud. RD **FA:** Hyperfluorescent lesions **ERG:** N/A | **Brain MRI:** N/A **CSF:** N/A **Serum Abs:** N/A | **Local Tx:** NSAIDs **IS Tx:** No **Onco Tx:** ChemoTx | Met. thyroid Ca + 3 months | Worsening | Death |
| M. Pefkianaki, 2015, UK | F, 62 | **Laterality:** Bilateral **Symptoms:** ↓ visual acuity **AC inflammation:** No **Fundus exam:** Pigmt. retinal lesions | **OCT:** Choroidal thickening **FA:** Hyperfluorescent lesions **ERG:** N/A | **Brain MRI:** Cerebellar lesion **CSF:** N/A **Serum Abs:** N/A | **Local Tx:** N/A **IS Tx:** No **Onco Tx:** ChemoTx | DLBCL + 12 months | Improvement | N/A |
| AS. Roblain, 2021, Belgium | F, 78 | **Laterality:** Bilateral **Symptoms:** ↓ visual acuity **AC inflammation:** No **Fundus exam:** Pigmt. retinal lesions | **OCT:** Exud. RD **FA:** Hyperfluorescent lesions **ERG:** N/A | **Brain MRI:** N/A **CSF:** N/A **Serum Abs:** N/A | **Local Tx:** No **IS Tx:** Steroids **Onco Tx:** ChemoTx | Met. colon ADK Anterior | Stable | Death |
| Z. Shalchi, 2014, UK | F, 48 | **Laterality:** Bilateral **Symptoms:** ↓ visual acuity **AC inflammation:** Yes **Fundus exam:** Pigmt. retinal lesions | **OCT:** Exud. RD **FA:** Hyperfluorescent lesions **ERG:** N/A | **Brain MRI:** N/A **CSF:** N/A **Serum Abs:** N/A | **Local Tx:** No **IS Tx:** N/A **Onco Tx:** Surgery | Metastatic ovarian adenocarcinoma - 12 months | N/A | N/A |
| Z. Shalchi, 2014, UK | F, 50 | **Laterality:** Bilateral **Symptoms:** ↓ visual acuity **AC inflammation:** No **Fundus exam:** Exud. RD | **OCT:** Exud. RD **FA:** Hyperfluorescent lesions **ERG:** N/A | **Brain MRI:** N/A **CSF:** N/A **Serum Abs:** N/A | **Local Tx:** No **IS Tx:** N/A **Onco Tx:** ChemoTx | Met. endom. ADK Recurrence | N/A | N/A |
| K. Tada, 2015, Japan | M, 72 | **Laterality:** Bilateral **Symptoms:** ↓ visual acuity **AC inflammation:** No **Fundus exam:** Exud. RD | **OCT:** Choroidal thickening **FA:** Hyperfluorescent lesions **ERG:** N/A | **Brain MRI:** N/A **CSF:** N/A **Serum Abs:** N/A | **Local Tx:** No **IS Tx:** No **Onco Tx:** Surgery | SCLC - 18 months | Worsening | Worsening |
| K. Tada, 2015, Japan | M, 50 | **Laterality:** Bilateral **Symptoms:** ↓ visual acuity **AC inflammation:** No **Fundus exam:** Pigmt. retinal lesions | **OCT:** N/A **FA:** Hyperfluorescent lesions **ERG:** N/A | **Brain MRI:** N/A **CSF:** N/A **Serum Abs:** N/A | **Local Tx:** No **IS Tx:** No **Onco Tx:** ChemoTx | SCLC Recurrence | Worsening | Worsening |
| A. Shirakia, 2018, Japan | M, 88 | **Laterality:** Bilateral **Symptoms:** ↓ visual acuity **AC inflammation:** No **Fundus exam:** Pigmt. retinal lesions | **OCT:** Exud. RD **FA:** N/A **ERG:** N/A | **Brain MRI:** N/A **CSF:** N/A **Serum Abs:** N/A | **Local Tx:** Steroids **IS Tx:** No **Onco Tx:** ChemoTx | Met. lung Ca Simultaneous | N/A | N/A |
| A. Shirakia, 2018, Japan | F, 46 | **Laterality:** Bilateral **Symptoms:** ↓ visual acuity **AC inflammation:** No **Fundus exam:** Exud. RD | **OCT:** RPE atrophy-thickening alternation **FA:** N/A **ERG:** N/A | **Brain MRI:** N/A **CSF:** N/A **Serum Abs:** N/A | **Local Tx:** Steroids **IS Tx:** No **Onco Tx:** ChemoTx | Met. ovarian Ca Simultaneous | Improvement | Recovery |
| A. Shirakia, 2018, Japan | M, 48 | **Laterality:** Bilateral **Symptoms:** ↓ visual acuity **AC inflammation:** No **Fundus exam:** Pigmt. retinal lesions | **OCT:** RPE atrophy-thickening alternation **FA:** N/A **ERG:** N/A | **Brain MRI:** N/A **CSF:** N/A **Serum Abs:** N/A | **Local Tx:** No **IS Tx:** No **Onco Tx:** N/A | No recurrence - 48 months | Improvement | N/A |

**Abs**: Antibodies, **AC**: Anterior chamber, **ADK**: Adenocarcinoma, **Beva**: Bevacizumab, **Ca**: Cancer, **CCC**: Clear cell carcinoma, **ChemoTx**: Chemotherapy, **Chor**: Choroidal, **CSF**: Cerebrospinal fluid, **DLBCL**: Diffuse large B-cell lymphoma, **ERG**: Electroretinogram, **Exud**: Exudative, **FA**: Fluorescein angiography, **H. therapy**: Hormone therapy, **ImmunoTx**: Immunotherapy, **IS Tx**: Immunosuppressive therapy, **MRI**: Magnetic resonance imaging, **N/A**: Not available, **NSAIDs**: Non-steroidal anti-inflammatory drugs, **OCT**: Optical coherence tomography, **Onco Tx**: Oncologic treatment, **Pigmt.**: Pigmented, **RadioTx**: Radiotherapy, **RD**: Retinal detachment, **RPE**: Retinal pigment epithelium, **SCC**: Squamous cell carcinoma, **SCLC**: Small cell lung cancer, **Steroids**: Corticosteroids, **Tx**: Treatment, **↓**: Decreased.
