# Supplementary material for: Paraneoplastic ocular syndromes: a systematic review of epidemiology, diagnosis and outcomes (2010–2023)
Source: J Ophthalmic Inflamm Infect. 2025 Sep 26;15:73. doi: 10.1186/s12348-025-00534-1 (PMC12474834; doi:10.1186/s12348-025-00534-1)
Supplement: Supplementary file 4 — Supplementary Material 4. [file 12348_2025_534_MOESM4_ESM.docx]

**Table 10: Characteristics of reported cases of paraneoplastic optic neuropathy**

| **Author, year, country** | **Sex, age** | **Ophthalmologic data** | **Ophthalmologic exams** | **Systemic workup** | **Treatment** | **Cancer, diagnosis timing** | **Visual outcome** | **Cancer outcome** |
| --- | --- | --- | --- | --- | --- | --- | --- | --- |
| E. Akbulut, 2021, Turkey | M, 65 | **Lat**: Bilateral **Symptoms**: Visual loss **VF**: N/A **ACI**: No **Fundus examination**: Optic disc edema | **OCT**: N/A **FA**: Peripapillary vessel hyperfluorescence **VEP**: N/A **ERG**: N/A | **MRI**: Optic nerve inflammation **CSF**: N/A **Serum Abs**: CRMP5 | **Local Tx**: No **IS Tx**: Corticosteroids **Onco Tx**: Chemotherapy | SCLC, + 6 months | Worsening | Death |
| G. Carboni, 2012, USA | M, 76 | **Lat**: Bilateral **Symptoms**: Visual loss **VF**: Enlarged blind spot **ACI**: No **Fundus examination**: Temporal optic disc atrophy | **OCT**: Normal **FA**: Peripapillary vessel hyperfluorescence **VEP**: Decreased amplitude **ERG**: Unilateral pathological | **MRI**: Normal **CSF**: N/A **Serum Abs**: Positive | **Local Tx**: No **IS Tx**: No **Onco Tx**: N/A | Prostate, - 48 months | N/A | N/A |
| T. Carette, 2021, Belgium | M, 70 | **Lat**: Bilateral **Symptoms**: Hemeralopia **VF**: N/A **ACI**: No **Fundus examination**: Optic disc edema | **OCT**: N/A **FA**: N/A **VEP**: Prolonged latency **ERG**: N/A | **MRI**: Normal **CSF**: Elevated protein **Serum Abs**: CRMP5 | **Local Tx**: No **IS Tx**: Corticosteroids **Onco Tx**: Chemotherapy | SCLC, Simultaneous | N/A | N/A |
| A. Durrani, 2016, USA | F, 67 | **Lat**: Bilateral **Symptoms**: Photopsia **VF**: N/A **ACI**: No **Fundus examination**: Peripapillary hemorrhage | **OCT**: Normal **FA**: Peripapillary vessel hyperfluorescence **VEP**: Prolonged latency **ERG**: Normal | **MRI**: Normal **CSF**: N/A **Serum Abs**: Hu | **Local Tx**: No **IS Tx**: Corticosteroids **Onco Tx**: N/A | Metastatic melanoma, Simultaneous | Improvement | N/A |
| A. Goudsmit, 2022, Belgium | M, 68 | **Lat**: Bilateral **Symptoms**: Photophobia **VF**: Altitudinal defects **ACI**: No **Fundus examination**: Optic disc edema | **OCT**: Normal **FA**: Normal **VEP**: N/A **ERG**: N/A | **MRI**: Optic nerve inflammation **CSF**: Normal **Serum Abs**: Amphiphysin | **Local Tx**: No **IS Tx**: Mycophenolate mofetil **Onco Tx**: Chemotherapy | Metastatic lung adenocarcinoma,  - 4 months | Worsening | Death |
| N. Igarashi, 2016, Japan | M, 68 | **Lat**: Bilateral **Symptoms**: Visual loss **VF**: Enlarged blind spot **ACI**: No **Fundus examination**: Left peripapillary atrophy | **OCT**: RNFL thickening **FA**: Peripapillary hyperfluorescence **VEP**: N/A **ERG**: N/A | **MRI**: Optic nerve inflammation **CSF**: Elevated protein **Serum Abs**: Negative | **Local Tx**: No **IS Tx**: Plasmapheresis **Onco Tx**: Chemotherapy | SCLC, Simultaneous | Recovery | N/A |
| M. Kubota, 2021, Japan | F, 76 | **Lat**: Bilateral **Symptoms**: Visual loss **VF**: Severe alteration **ACI**: No **Fundus examination**: Hyalitis | **OCT**: Normal **FA**: N/A **VEP**: Decreased amplitude **ERG**: Normal | **MRI**: Optic nerve inflammation **CSF**: Normal **Serum Abs**: CRMP5 | **Local Tx**: No **IS Tx**: Corticosteroids **Onco Tx**: Radiotherapy | Metastatic lung adenocarcinoma,  + 2 months | Improvement | Death |
| C. Lee, 2020, South Korea | M, 60 | **Lat**: Bilateral **Symptoms**: Floaters **VF**: Altitudinal defects **ACI**: No **Fundus examination**: Disc pallor | **OCT**: RNFL thickening **FA**: Peripapillary hyperfluorescence **VEP**: Decreased amplitude **ERG**: Normal | **MRI**: Normal **CSF**: N/A **Serum Abs**: CRMP5 | **Local Tx**: No **IS Tx**: Corticosteroids **Onco Tx**: Targeted therapy | Pancreatic adenocarcinoma, - 4 months | Improvement | N/A |
| O. Passarin, 2014, Switzerland | M, 80 | **Lat**: Unilateral **Symptoms**: Photopsia **VF**: N/A **ACI**: Yes **Fundus examination**: Normal | **OCT**: N/A **FA**: Peripapillary hyperfluorescence **VEP**: Prolonged latency **ERG**: N/A | **MRI**: Normal **CSF**: Elevated protein **Serum Abs**: Negative | **Local Tx**: No **IS Tx**: Corticosteroids **Onco Tx**: Chemotherapy | SCLC, Simultaneous | Improvement | Worsening |
| JA. Micieli, 2017, Canada | F, 57 | **Lat**: Bilateral **Symptoms**: Visual loss **VF**: Inferior defects **ACI**: No **Fundus examination**: Optic disc edema | **OCT**: N/A **FA**: N/A **VEP**: N/A **ERG**: N/A | **MRI**: Normal **CSF**: N/A **Serum Abs**: PCA2 | **Local Tx**: No **IS Tx**: N/A **Onco Tx**: Radiotherapy | SCLC, + 1 month | Stable | Recovery |
| J. Nadal Bosch, 2023, USA | F, 40 | **Lat**: Bilateral **Symptoms**: Visual loss **VF**: Impossible **ACI**: No **Fundus examination**: Hyalitis | **OCT**: N/A **FA**: N/A **VEP**: N/A **ERG**: N/A | **MRI**: Optic nerve inflammation **CSF**: N/A **Serum Abs**: Negative | **Local Tx**: No **IS Tx**: Corticosteroids **Onco Tx**: Chemotherapy | Metastatic cervical cancer,  - 1 month | Improvement | N/A |
| M. Nakajima, 2018, Japan | F, 61 | **Lat**: Unilateral **Symptoms**: Visual loss **VF**: N/A **ACI**: No **Fundus examination**: Optic disc edema | **OCT**: N/A **FA**: N/A **VEP**: Prolonged latency **ERG**: Pathologic (cones and rods) | **MRI**: Optic nerve inflammation **CSF**: Normal **Serum Abs**: CRMP5 | **Local Tx**: No **IS Tx**: Plasmapheresis **Onco Tx**: Chemotherapy | SCLC, + 9 months | Improvement | N/A |
| R. Paul, 2014, India | F, 40 | **Lat**: Unilateral **Symptoms**: Visual loss **VF**: N/A **ACI**: No **Fundus examination**: Optic disc edema | **OCT**: N/A **FA**: N/A **VEP**: N/A **ERG**: N/A | **MRI**: Optic nerve inflammation **CSF**: Elevated protein **Serum Abs**: Hu | **Local Tx**: No **IS Tx**: No **Onco Tx**: Chemotherapy | Pancreatic adenocarcinoma, + 2 months | Worsening | Death |
| S. Schoenberger, 2012, USA | F, 67 | **Lat**: Unilateral **Symptoms**: Visual loss **VF**: Tubular **ACI**: No **Fundus examination**: Optic disc edema | **OCT**: N/A **FA**: Peripapillary hyperfluorescence **VEP**: Prolonged latency **ERG**: Normal | **MRI**: N/A **CSF**: N/A **Serum Abs**: Not done | **Local Tx**: No **IS Tx**: Corticosteroids **Onco Tx**: No | Metastatic melanoma,  + 2 months | Stable | N/A |
| R. Yan, 2023, China | F, 59 | **Lat**: Bilateral **Symptoms**: Scotoma **VF**: Enlarged blind spot **ACI**: Yes **Fundus examination**: Hyalitis | **OCT**: Peripapillary detachment **FA**: N/A **VEP**: N/A **ERG**: N/A | **MRI**: Optic nerve inflammation **CSF**: Elevated protein **Serum Abs**: CRMP5 | **Local Tx**: N/A **IS Tx**: Corticosteroids **Onco Tx**: N/A | SCLC, + 1 month | N/A | N/A |
| R. Yan, 2023, China | M, 69 | **Lat**: Bilateral **Symptoms**: Photopsia **VF**: Central scotoma **ACI**: No **Fundus examination**: Optic disc edema | **OCT**: Peripapillary detachment **FA**: N/A **VEP**: N/A **ERG**: N/A | **MRI**: Optic nerve inflammation **CSF**: Normal **Serum Abs**: CRMP5 | **Local Tx**: N/A **IS Tx**: Mycophenolate mofetil **Onco Tx**: N/A | SCLC, + 2 months | N/A | N/A |
| R. Yan, 2023, China | F, 53 | **Lat**: Bilateral **Symptoms**: Visual loss **VF**: Central scotoma **ACI**: No **Fundus examination**: Hyalitis | **OCT**: Retinal detachment **FA**: N/A **VEP**: N/A **ERG**: N/A | **MRI**: Optic nerve inflammation **CSF**: Elevated protein **Serum Abs**: CRMP5 | **Local Tx**: N/A **IS Tx**: Plasmapheresis **Onco Tx**: N/A | Papillary thyroid carcinoma,  + 4 months | N/A | N/A |
| R. Yan, 2023, China | F, 61 | **Lat**: Bilateral **Symptoms**: Visual loss **VF**: Central scotoma **ACI**: No **Fundus examination**: Optic disc edema | **OCT**: IS/OS junction loss **FA**: N/A **VEP**: N/A **ERG**: N/A | **MRI**: Optic nerve inflammation **CSF**: N/A **Serum Abs**: CRMP5 | **Local Tx**: N/A **IS Tx**: N/A **Onco Tx**: N/A | Thymoma,  + 10 months | N/A | N/A |
| J. Yap, 2021, Malaysia | M, 45 | **Lat**: Bilateral **Symptoms**: Visual loss **VF**: Impossible **ACI**: No **Fundus examination**: Hyalitis | **OCT**: N/A **FA**: N/A **VEP**: Decreased amplitude **ERG**: N/A | **MRI**: N/A **CSF**: Normal **Serum Abs**: CRMP5 | **Local Tx**: No **IS Tx**: N/A **Onco Tx**: Surgery, Chemotherapy | Metastatic lung adenocarcinoma, Simultaneous | Improvement | Death |

**ACI**: Anterior chamber inflammation, **Ab**: Antibody, **CRMP5**: Collapsin Response Mediator Protein 5, **CSF**: Cerebrospinal fluid, **Dx**: Diagnosis, **ERG**: Electroretinogram, **FA**: Fluorescein angiography, **Fundus**: Fundus examination, **IS Tx**: immunosuppressive treatment, **Lat**: Laterality, **Local Tx**: Local treatment, **MRI**: Magnetic resonance imaging, **N/A**: Not available, **OCT**: Optical coherence tomography, **Onco** **Tx**: Oncologic treatment, **Tx**: Treatment, **VEP**: Visual evoked potentials, **VF**: Visual field.
