# Supplementary material for: Paraneoplastic ocular syndromes: a systematic review of epidemiology, diagnosis and outcomes (2010–2023)
Source: J Ophthalmic Inflamm Infect. 2025 Sep 26;15:73. doi: 10.1186/s12348-025-00534-1 (PMC12474834; doi:10.1186/s12348-025-00534-1)
Supplement: Supplementary file 5 — Supplementary Material 5. [file 12348_2025_534_MOESM5_ESM.docx]

**Table 8: Characteristics of reported cases of acute exudative polymorphous paraneoplastic vitelliform maculopathy**

| **Author, year, country** | **Sex, age** | **Ophthalmologic data** | **Ophthalmologic exams** | **Systemic workup** | **Treatment** | **Cancer, diagnosis timing** | **Visual outcome** | **Cancer outcome** |
| --- | --- | --- | --- | --- | --- | --- | --- | --- |
| L. Koreen, 2011, United States | M, 50 | **Lat:** Bilateral **Sx:** Visual acuity loss **ACI:** No **Fundus exam:** Subretinal fluid, RPE lesion | **OCT:** Serous retinal detachment, subretinal deposits **FA:** Hyperfluorescent lesions **ERG:** Pathologic (cones and rods) | **Serum Abs:** PRDX3 | **Local Tx:** No **IS Tx:** Corticosteroids **Onco Tx:** Temozolomide | Metastatic melanoma +4 months | Recovery | Recovery |
| J. Gao, 2020, United States | M, 58 | **Lat:** Bilateral **Sx:** Visual acuity loss **ACI:** No **Fundus exam:** Macular subretinal lesions | **OCT:** Serous retinal detachment, subretinal deposits **FA:** Hyperfluorescent lesions **ERG:** N/A | **Serum Abs:** N/A | **Local Tx:** Intravitreal corticosteroids, intravitreal methotrexate **IS Tx:** Corticosteroids **Onco Tx:** Dabrafenib Trametinib | Metastatic melanoma -12 months | Improvement | Recovery |
| A. Laouani, 2022, Belgium | M, 32 | **Lat:** Bilateral **Sx:** Visual acuity loss **ACI:** Yes **Fundus exam:** Vitelliform-like lesions | **OCT:** Serous retinal detachment **FA:** Hyperfluorescent lesions **ERG:** N/A | **Serum Abs:** N/A | **Local Tx:** N/A **IS Tx:** N/A **Onco Tx:** Rituximab | Marginal zone lymphoma +6 months | N/A | N/A |
| L. Grunwald, 2011, United States | F, 69 | **Lat:** Bilateral **Sx:** Visual acuity loss, photopsia, hemeralopia **ACI:** No **Fundus exam:** Vitelliform-like lesions | **OCT:** Serous retinal detachment, subretinal deposits **FA:** Hypofluorescent lesions **ERG:** N/A | **Serum Abs:** N/A | **Local Tx:** N/A **IS Tx:** N/A **Onco Tx:** Chemotherapy | Metastatic lung cancer -24 months | N/A | N/A |
| ME. Aronow, 2012, United States | M, 80 | **Lat:** Bilateral **Sx:** Visual acuity loss **ACI:** No **Fundus exam:** Macular subretinal lesions | **OCT:** Outer plexiform layer lesion **FA:** Hypofluorescent lesions **ERG:** Pathologic (cones and rods) | **Serum Abs:** CA 2 | **Local Tx:** No **IS Tx:** No **Onco Tx:** Chemotherapy | Metastatic melanoma -12 months | N/A | Death |
| PG. Meier, 2015, Switzerland | F, 41 | **Lat:** Bilateral **Sx:** Visual acuity loss **ACI:** No **Fundus exam:** Macular subretinal lesions | **OCT:** Serous retinal detachment, subretinal deposits **FA:** Hypofluorescent lesions **ERG:** Pathologic (cones and rods) | **Serum Abs:** PEDF | **Local Tx:** No **IS Tx:** No **Onco Tx:** Chemotherapy | Metastatic melanoma Simultaneous | Improvement | N/A |
| H. Krema, 2010, Canada | M, 58 | **Lat:** Bilateral **Sx:** Visual acuity loss **ACI:** No **Fundus exam:** Macular subretinal lesions | **OCT:** Outer layer lesions **FA:** Hypofluorescent lesions **ERG:** Normal | **Serum Abs:** Negative | **Local Tx:** No **IS Tx:** No **Onco Tx:** Chemotherapy | Metastatic melanoma +2 months | Stable | N/A |
| M. Ouederni, 2022, Tunisia | M, 59 | **Lat:** Bilateral **Sx:** Visual acuity loss **ACI:** No **Fundus exam:** Multifocal macular lesions | **OCT:** Lesions between RPE and photoreceptor layer **FA:** Hypofluorescent lesions **ERG:** N/A | **Serum Abs:** N/A | **Local Tx:** No **IS Tx:** No **Onco Tx:** Chemotherapy | Metastatic spread of squamous cell carcinoma Simultaneous | N/A | N/A |
| M. Rahimi, 2017, United States | M, 44 | **Lat:** Bilateral **Sx:** Visual acuity loss and photophobia **ACI:** No **Fundus exam:** Vitelliform macular lesions | **OCT:** Serous retinal detachment and RPE deposits **FA:** Hypofluorescent lesions **ERG:** Normal | **Serum Abs:** N/A | **Local Tx:** No **IS Tx:** N/A **Onco Tx:** Targeted therapy | Metastatic melanoma -2 months | Worsening | N/A |
| HS. Sandhu, 2020, United States | F, 55 | **Lat:** Bilateral **Sx:** Visual acuity loss **ACI:** No **Fundus exam:** Subretinal deposits | **OCT:** Serous retinal detachment **FA:** Normal **ERG:** N/A | **Serum Abs:** N/A | **Local Tx:** Corticosteroids **IS Tx:** No **Onco Tx:** Targeted therapy | Metastatic melanoma -10 months | Recovery | Death |
| A. El Ameen, 2016, France | M, 65 | **Lat:** Unilateral **Sx:** Visual acuity loss **ACI:** No **Fundus exam:** Subretinal deposits | **OCT:** Serous retinal detachment and RPE deposits **FA:** Hypofluorescent lesions **ERG:** N/A | **Serum Abs:** N/A | **Local Tx:** No **IS Tx:** Corticosteroids **Onco Tx:** Velcade | IgG multiple myeloma Simultaneous | Recovery | Improvement |
| I. Rusu, 2014, United States | F, 58 | **Lat:** Bilateral **Sx:** Visual acuity loss **ACI:** No **Fundus exam:** RPE lesions | **OCT:** Serous retinal detachment and RPE deposits **FA:** Hypofluorescent lesions **ERG:** N/A | **Serum Abs:** N/A | **Local Tx:** Anti-VEGF **IS Tx:** Corticosteroids **Onco Tx:** Velcade | IgM multiple myeloma -8 months | Recovery | Improvement |
| I. Rusu, 2014, United States | M, 55 | **Lat:** Bilateral **Sx:** Visual acuity loss **ACI:** No **Fundus exam:** Subretinal deposits | **OCT:** RPE deposits **FA:** Normal **ERG:** Pathologic (cones and rods) | **Serum Abs:** N/A | **Local Tx:** No **IS Tx:** Plasmapheresis **Onco Tx:** Hematopoietic stem cell transplant | Multiple myeloma +24 months | Recovery | Recovery |
| I. Rusu, 2014, United States | M, 62 | **Lat:** Bilateral **Sx:** Visual acuity loss **ACI:** No **Fundus exam:** Serous retinal detachment | **OCT:** RPE deposits **FA:** N/A **ERG:** N/A | **Serum Abs:** N/A | **Local Tx:** No **IS Tx:** Corticosteroids **Onco Tx:** Velcade | IgM multiple myeloma N/A | Recovery | Improvement |
| JM. Khan, 2010, United Kingdom | F, 73 | **Lat:** Bilateral **Sx:** Visual acuity loss **ACI:** No **Fundus exam:** Serous retinal detachment and RPE lesions | **OCT:** Serous retinal detachment **FA:** Hypofluorescent lesions **ERG:** Normal | **Serum Abs:** N/A | **Local Tx:** No **IS Tx:** Corticosteroids **Onco Tx:** Melphalan Thalidomide | IgG multiple myeloma +24 months | Stable | Improvement |
| C. Bianciotto, 2010, United States | M, 74 | **Lat:** Bilateral **Sx:** Visual acuity loss **ACI:** No **Fundus exam:** Serous retinal detachment | **OCT:** Serous retinal detachment **FA:** N/A **ERG:** Normal | **Serum Abs:** IRBP | **Local Tx:** No **IS Tx:** No **Onco Tx:** Chemotherapy, radiotherapy | Metastatic melanoma -3 months | N/A | N/A |

**Abs**: Antibodies, **ACI**: Anterior chamber inflammation, **ERG**: Electroretinogram, **FA**: Fluorescein angiography, **Fundus** **exam**: Fundus examination, **IS** **Tx**: Immunosuppressive treatment, **Lat**: Laterality, **Local Tx**: Local treatment, **N/A**: Not available, **OCT**: Optical coherence tomography, **Onco** **Tx**: Oncologic treatment, **Sx**: Symptom
