# Supplementary material for: Paraneoplastic ocular syndromes: a systematic review of epidemiology, diagnosis and outcomes (2010–2023)
Source: J Ophthalmic Inflamm Infect. 2025 Sep 26;15:73. doi: 10.1186/s12348-025-00534-1 (PMC12474834; doi:10.1186/s12348-025-00534-1)
Supplement: Supplementary file 6 — Supplementary Material 6. [file 12348_2025_534_MOESM6_ESM.docx]

**Table 9: Characteristics of reported cases of paraneoplastic uveitis**

| **Author, year, country** | **Sex, age** | **Ophthalmologic data** | **Ophthalmologic exams** | **Systemic workup** | **Treatment** | **Cancer, diagnosis timing** | **Visual outcome** | **Cancer outcome** |
| --- | --- | --- | --- | --- | --- | --- | --- | --- |
| A Alshamrani, 2020, Saudi Arabia | M, 25 | **Laterality**: Bilateral **Symptoms**: Decreased visual acuity **AC inflammation**: Yes **Fundus**: Hyalitis | **OCT**: Ellipsoid and interdigitation zone lesions **FA**: Hypofluorescent lesions **Anterior chamber tap**: N/A **Vitrectomy**: N/A | **Brain MRI**: Normal **CSF analysis**: N/A **Serum Abs**: N/A | **Local Tx**: Corticosteroids **IS Tx**: Corticosteroids **Onco Tx**: Rituximab | Hodgkin lymphoma, Simultaneous | Recovery | Recovery |
| EJ. Casswell, 2015, United Kingdom | F, 52 | **Laterality**: Bilateral **Symptoms**: Ocular redness **AC inflammation**: Granulomatous **Fundus**: Chorioretinal lesions | **OCT**: Foveal atrophy **FA**: Diffuse capillary leakage at optic disc **Anterior chamber tap**: N/A **Vitrectomy**: N/A | **Brain MRI**: Normal **CSF analysis**: Normal **Serum Abs**: CRMP5 | **Local Tx**: Corticosteroids **IS Tx**: Corticosteroids **Onco Tx**: Chemotherapy | Metastatic small-cell lung carcinoma, Simultaneous | N/A | Recovery |
| L. Hernández-Bel, 2019, Spain | M, 59 | **Laterality**: Bilateral **Symptoms**: Photophobia **AC inflammation**: No **Fundus**: Hyalitis | **OCT**: N/A **FA**: Hyperfluorescence of optic nerve **Anterior chamber tap**: N/A **Vitrectomy**: N/A | **Brain MRI**: Normal **CSF analysis**: N/A **Serum Abs**: Amphiphysin | **Local Tx**: N/A **IS Tx**: Corticosteroids **Onco Tx**: Chemotherapy | Small-cell lung carcinoma, Simultaneous | Recovery | Recovery |
| E. Hughes, 2016, Ireland | M, 51 | **Laterality**: Unilateral **Symptoms**: Decreased visual acuity **AC inflammation**: No **Fundus**: Hyalitis | **OCT**: N/A **FA**: N/A **Anterior chamber tap**: N/A **Vitrectomy**: N/A | **Brain MRI**: Normal **CSF analysis**: Normal **Serum Abs**: Hu | **Local Tx**: N/A **IS Tx**: Corticosteroids **Onco Tx**: Chemotherapy | NK/T-cell lymphoma,  +10 months | N/A | N/A |
| L. Iannetti, 2010, Italy | F, 76 | **Laterality**: Bilateral **Symptoms**: Decreased visual acuity **AC inflammation**: N/A **Fundus**: Papillitis | **OCT**: Normal **FA**: Hyperfluorescent lesions **Anterior chamber tap**: N/A **Vitrectomy**: N/A | **Brain MRI**: N/A **CSF analysis**: N/A **Serum Abs**: CRMP5 | **Local Tx**: Corticosteroids **IS Tx**: Corticosteroids **Onco Tx**: Chemotherapy | Metastatic ductal breast carcinoma, Simultaneous | Recovery | N/A |
| RK. Khanna, 2018, France | F, 49 | **Laterality**: Unilateral **Symptoms**: Decreased visual acuity **AC inflammation**: Yes **Fundus**: Panuveitis | **OCT**: Subretinal detachment **FA**: Hyperfluorescent lesions **Anterior chamber tap**: N/A **Vitrectomy**: N/A | **Brain MRI**: Normal **CSF analysis**: N/A **Serum Abs**: Negative | **Local Tx**: N/A **IS Tx**: Corticosteroids **Onco Tx**: Chemotherapy | MALT lymphoma, Simultaneous | Worsening | Improvement |
| G. Şatırtav, 2016, Turkey | M, 79 | **Laterality**: Unilateral **Symptoms**: Decreased visual acuity **AC inflammation**: Hypopyon **Fundus**: Hyalitis | **OCT**: Macular edema **FA**: Peripheral and optic nerve vascular leakage **Anterior chamber tap**: N/A **Vitrectomy**: N/A | **Brain MRI**: Normal **CSF analysis**: N/A **Serum Abs**: N/A | **Local Tx**: Corticosteroids **IS Tx**: N/A **Onco Tx**: Chemotherapy | Urothelial bladder carcinoma,  +9 months | Recovery | Recovery |
| EK. Yigitaslan, 2021, Turkey | M, 70 | **Laterality**: Bilateral **Symptoms**: Ocular redness **AC inflammation**: Yes **Fundus**: Choroidal lesions | **OCT**: Normal **FA**: Ischemia **Anterior chamber tap**: N/A **Vitrectomy**: N/A | **Brain MRI**: N/A **CSF analysis**: N/A **Serum Abs**: N/A | **Local Tx**: N/A **IS Tx**: Corticosteroids **Onco Tx**: Surgery + Chemotherapy | Small-cell lung carcinoma, Simultaneous | Recovery | Death |
| M. Zako, 2022, Japan | M, 18 | **Laterality**: Bilateral **Symptoms**: Decreased visual acuity **AC inflammation**: No **Fundus**: Hyalitis | **OCT**: Optic disc edema **FA**: N/A **Anterior chamber tap**: N/A **Vitrectomy**: N/A | **Brain MRI**: Lesion in third ventricle **CSF analysis**: N/A **Serum Abs**: N/A | **Local Tx**: N/A **IS Tx**: Corticosteroids **Onco Tx**: Surgery, Chemotherapy, Radiotherapy | Thymic and pineal germinoma, Simultaneous | Recovery | N/A |
| A. Pierru, 2013, France | F, 64 | **Laterality**: Bilateral **Symptoms**: Floaters **AC inflammation**: Yes **Fundus**: Venous vasculitis, papilledema | **OCT**: Normal **FA**: Venous vasculitis, papilledema **Anterior chamber tap**: N/A **Vitrectomy**: N/A | **Brain MRI**: Normal **CSF analysis**: Normal **Serum Abs**: N/A | **Local Tx**: Corticosteroids **IS Tx**: Corticosteroids **Onco Tx**: Surgery, Chemotherapy, Radiotherapy | Papillary thyroid carcinoma, +1 month | Recovery | Recovery |
| HE. Moss, 2010, United States | M, 60 | **Laterality**: Bilateral **Symptoms**: Decreased visual acuity, ocular pain **AC inflammation**: No **Fundus**: Hyalitis, papilledema | **OCT**: N/A **FA**: Normal **Anterior chamber tap**: N/A **Vitrectomy**: N/A | **Brain MRI**: Normal **CSF analysis**: Normal **Serum Abs**: CRMP5 | **Local Tx**: N/A **IS Tx**: Corticosteroids **Onco Tx**: N/A | Small-cell lung carcinoma, +3 months | Improvement | Improvement |

**Abs**: Antibodies, **AC**: Anterior chamber, **CRMP5**: Collapsin Response Mediator Protein 5, **CSF**: Cerebrospinal fluid, **FA**: Fluorescein angiography, **IS**: Immunosuppressive, **IS Tx**: Immunosuppressive treatment, **Local Tx**: Local treatment, **MALT**: Mucosa-Associated Lymphoid, Tissue, **MRI**: Magnetic resonance imaging, **N/A**: Non available, **OCT**: Optical coherence tomography, **Onco Tx**: Oncologic treatment
